# Targeting PSMD14 enhances immunotherapy efficacy by promoting PD-L1 degradation and reshaping the tumor microenvironment in breast cancer

**DOI:** 10.1186/s13046-026-03710-w

**Published:** 2026-04-14

**Authors:** Shichao Wen, Yuhan Liu, Qi Liu, Liqian Su, Yuhua Wang, Yiqiu Ma, Jingxuan Wang

**Affiliations:** 1 Department of Medical Oncology, Harbin Medical University Cancer Hospital, 150 Haping Road, Harbin, 150081 China; 2 Precision Medicine Center, Harbin Medical University Cancer Hospital, 150 Haping Road, Harbin, 150081 China

**Keywords:** PSMD14, PD-L1, Deubiquitination, Immunotherapy, Breast cancer, Tumor microenvironment

## Abstract

**Background:**

Programmed death-1 (PD-1) and programmed death-ligand 1 (PD-L1) inhibitors have shown encouraging clinical efficacy in breast cancer, primarily by modulating the tumor microenvironment (TME). However, achieving durable clinical responses remains a major challenge. Although the deubiquitinating enzyme 26S proteasome non-ATPase regulatory subunit 14 (PSMD14) is known to exert oncogenic functions in various cancers, its potential role in regulating tumor immune evasion remains unclear. This study investigates how PSMD14 regulates PD-L1 and reshapes the TME, with the goal of clarifying its impact on immune regulation in breast cancer.

**Methods:**

PSMD14 expression and its prognostic significance in breast cancer were analyzed using public databases and clinical samples. The correlation between PSMD14 and PD-L1 was validated by immunohistochemistry (IHC), western blot, and flow cytometry. The mechanism by which PSMD14 stabilizes PD-L1 through deubiquitination and their interaction was investigated using cycloheximide chase assays, co-immunoprecipitation (Co-IP), and ubiquitination assays. The impact of PSMD14 on the TME and its influence on immunotherapy efficacy were evaluated using T cell cytotoxicity assays, syngeneic mouse models, and flow cytometry analyses.

**Results:**

PSMD14 was highly expressed in breast cancer and correlated with poor patient prognosis. Mechanistically, PSMD14 stabilized PD-L1 by interacting with its intracellular domain and removing its K48-linked polyubiquitin chains, thereby inhibiting proteasomal degradation. Inhibition of PSMD14 enhanced antigen presentation and CD8⁺ T cell activation, reduced the accumulation of regulatory T cells (Tregs) and myeloid-derived suppressor cells (MDSCs), and altered macrophage polarization. These changes collectively improved the response to immunotherapy in breast cancer.

**Conclusion:**

This study identifies PSMD14 as a critical regulator of immune responses in breast cancer. Targeting PSMD14 enhances the effectiveness of immunotherapy by promoting PD-L1 degradation and remodeling the TME, offering a potential strategy to improve clinical outcomes.

**Supplementary Information:**

The online version contains supplementary material available at 10.1186/s13046-026-03710-w.

## Background

Breast cancer is one of the most commonly diagnosed malignancies in women, and its treatment has increasingly shifted toward precision medicine. Immune checkpoint inhibitors (ICIs) targeting programmed death-1 (PD-1) and programmed death-ligand 1 (PD-L1) have shown promising clinical efficacy. By blocking the interaction between PD-1 and PD-L1, these inhibitors restore T cell function and prevent immune evasion [1, 2]. However, durable responses remain limited in a substantial proportion of patients, largely due to the immunosuppressive tumor microenvironment (TME). Accumulating evidence suggests that the effectiveness of immunotherapy is influenced by multiple factors within the TME, including PD-L1 expression, immune cell infiltration, antigen presentation capacity, and cytokine signaling [3]. A deeper understanding of immune checkpoints and TME regulation is essential for improving immunotherapeutic strategies and converting “cold” tumors into “hot” ones.

Ubiquitination is a reversible post-translational modification (PTM) that regulates protein stability and function [4]. In an ATP-dependent process, the E1 enzyme activates ubiquitin and transfers it to an E2 conjugating enzyme, followed by E3 ligase-mediated recruitment to facilitate substrate ubiquitination [5–7]. The balance between ubiquitination and deubiquitination is maintained by deubiquitinating enzymes (DUBs) [8, 9]. DUB inhibitors are now being explored in clinical settings; for example, the USP1 inhibitor has entered clinical trials as monotherapy or in combination with PARP inhibitors for advanced malignancies [10]. Beyond their role in protein turnover, DUBs are increasingly recognized as important regulators of immune responses. For instance, USP2 deubiquitinates and stabilizes PD-L1, thereby promoting immune evasion by suppressing CD8⁺ T cell activation and the release of cytotoxic factors [11]. Therefore, elucidating the relationship between DUBs and immune regulation is essential for advancing clinical translation and developing synergistic therapeutic strategies.

The 26S proteasome non-ATPase regulatory subunit 14 (PSMD14), a member of the JAMM metalloprotease family and a critical component of the 19S regulatory particle of the 26S proteasome, plays a pivotal role in regulating protein stability, cell cycle progression, and apoptosis [12]. PSMD14 is overexpressed in various malignancies, such as esophageal squamous cell carcinoma, pancreatic cancer, and breast cancer [13–15]. In breast cancer, PSMD14 increases estrogen signals by stabilizing ERα through deubiquitination [16], and the PSMD14/E2F1 axis has been shown to suppress ferroptosis and promote the proliferation of triple-negative breast cancer (TNBC) by upregulating CENPF [17]. Despite these findings, it remains unclear whether PSMD14 contributes to immune evasion of breast cancer by modulating the TME.

In this study, we found that PSMD14 enhances PD-L1 expression in breast cancer. Mechanistically, PSMD14 interacted with the PD-L1 intracellular domain and stabilized its level by inhibiting K48-linked polyubiquitination. Notably, our investigation of the TME revealed that inhibition of PSMD14 enhanced CD8⁺ T cell-mediated cytotoxicity by increasing cytokine release; reduced the accumulation of immunosuppressive cells, and altered macrophage polarization. This multifaceted modulation of the TME ultimately enhanced immune responses in breast cancer. Taken together, our findings identify PSMD14 as a promising target for improving immunotherapy in breast cancer.

## Methods

### Breast tissue specimens

A total of 90 breast cancer tissue specimens were collected from patients treated at Harbin Medical University Cancer Hospital. Detailed clinicopathological characteristics are summarized in Table S1 and Table S2. Survival analysis was performed using follow-up data. Overall survival (OS) was defined as the time from the date of surgery to death from any cause or the last follow-up. Treatment response was evaluated according to the immune response Evaluation Criteria in Solid Tumors (iRECIST) [18]. Only patients with complete clinicopathological information and available survival data were included in the analysis. This study was approved by the Institutional Ethics Committee of Harbin Medical University Cancer Hospital (No. KY2023-02).

### Cell culture

All cell lines were obtained from the National Collection of Authenticated Cell Cultures of Chinese Academy of Sciences. MDA-MB-231 cells were cultured in L15 medium (Gibco), while MDA-MB-468 and HEK-293T cells were grown in DMEM (Gibco). BT-549 and murine 4T1 cells were maintained with RPMI-1640 medium (Gibco). All media were supplemented with 10% fetal bovine serum (PAN-Biotech) and 1% penicillin-streptomycin (Beyotime). Cells were maintained at 37 °C in a humidified environment with or without 5% CO₂.

### Plasmids and transfection

Plasmids encoding the catalytically inactive PSMD14 mutant (H113Q), full-length and ΔC-tail His-PD-L1, and HA-ubiquitin mutants were obtained from MiaoLing Bio. Lentiviral shRNAs and overexpression constructs targeting human and murine PSMD14 were also provided by MiaoLing Bio. PD-L1 small interfering RNAs (siRNAs) were acquired from GenePharma. All constructs were verified by DNA sequencing (Table S3). Transfection of plasmids and siRNAs was performed using the jetPRIME reagent (Polyplus) according to the manufacturer’s instructions. Lentiviral transduction was conducted at optimal multiplicity of infection (MOI) values, followed by puromycin selection to establish stable cell lines.

### Immunohistochemistry (IHC)

Formalin-fixed and paraffin-embedded tissue sections were subjected to deparaffinization for antigen retrieval, followed by blocking of endogenous peroxidase and non-specific binding sites. After incubating the primary antibody at 4 °C overnight, an HRP-conjugated second antibody and DAB were used to produce signals, followed by hematoxylin counterstaining. The stained sections were then dehydrated, mounted, and examined under a microscope. Staining was scored independently by two pathologists based on the percentage of positive cells and staining intensity, with low expression defined as a score < 6 and high expression as a score ≥ 6.

### Western blot and co-immunoprecipitation (Co-IP) assays

Cellular lysis was conducted with RIPA buffer on ice for 30 min, and the lysates were collected by centrifugation at 10,000 × g for 10 min at 4 °C. The BCA method (Beyotime) was used to quantify the protein concentration. Equal amounts of protein were separated by 6–15% SDS-PAGE gels (Epizyme) and transferred onto PVDF membranes (Millipore). After blocking with 5% skim milk for 2 h at room temperature, membranes were probed with the primary antibody overnight at 4 °C, followed by incubation with HRP-conjugated secondary antibodies for 1 h at room temperature. Protein signals were visualized using a Tanon imaging system. Table S4 contains comprehensive information about antibodies.

For Co-IP, cells were lysed in IP lysis buffer on ice for 30 min, and supernatants were collected after centrifugation (12,000 × g, 10 min, 4 °C). Following the manufacturer’s instructions, antibodies were pre-incubated with Protein A/G agarose beads at 4 °C for 2 h, followed by incubation with cell lysates overnight at 4 °C. Immunoprecipitated complexes were then eluted and analyzed by SDS-PAGE.

### qRT-PCR assay

Total RNA was isolated using TRNzol reagent (Tiangen Bio), and its concentration and purity were measured by NanoDrop 2000 (Thermo Fisher Scientific). Complementary DNA (cDNA) was synthesized using the FastKing gDNA Dispelling RT SuperMix (Tiangen Bio). qRT-PCR was performed using SuperReal PreMix Plus (Tiangen Bio) and StepOnePlus System (Thermo Fisher). GAPDH was used as the internal control. Primer sequences are listed in Table S5.

### EdU assay

Cell proliferation was evaluated using an EdU assay kit (Abbkine). Treated cells were seeded in 96-well plates and cultured for 24 h, followed by incubation with 20 µM EdU for 2 h at 37 °C. Cells were then fixed with 4% paraformaldehyde, permeabilized with 0.5% Triton X-100, and stained with the Click-iT reaction mixture in the dark. Nuclei were counterstained with Hoechst 33,342. EdU-positive cells were visualized using an inverted fluorescence microscope (Leica) and quantified with ImageJ software.

### Cell counting kit-8 (CCK-8) assay

Cell proliferation was assessed using a SuperKine Maximum Sensitivity Cell Counting Kit-8 (Abbkine). Cells were seeded at a density of 3 × 10^3^ cells per well in 96-well plates. At 0, 24, 48, 72, and 96 h, CCK-8 reagent was added and incubated for 2 h at 37 °C in the dark. Absorbance was detected using a BioTek microplate reader at 450 nm.

### Protein half-life assay

Cells were treated with cycloheximide (CHX, 50 µg/mL) upon reaching appropriate density (70%-80%). Subsequently, cells were collected at 0, 4, 8, 12, and 16 h for western blot analysis.

### Immunofluorescence (IF) staining

For IF experiment, cells on coverslips were cultured for 24 h. When cells reached an appropriate density, they were fixed with 4% paraformaldehyde at room temperature for 20 min, followed by permeabilization with 0.25% Triton X-100 and blocking with 5% BSA. The coverslips were then incubated with primary antibodies overnight at 4 °C and secondary antibodies for 1 h in the dark. Nuclei were counterstained with DAPI. Images were captured using a confocal microscope, and the fluorescence intensity and localization were analyzed with ImageJ software.

### Molecular docking

The structure of PSMD14 was predicted using AlphaFold3, while the PD-L1 intracellular domain (ICD) structure was obtained from the RCSB Protein Data Bank (PDB ID: 6L8R) (https://www.rcsb.org). Molecular docking of PSMD14 and PD-L1 ICD was performed using the HDOCK program to generate potential interaction models. Structural visualization and analysis were carried out using PyMOL.

### T cell-mediated cytotoxicity assay

Human peripheral blood mononuclear cells (PBMCs) were isolated from healthy human blood by lymphocyte separation medium (TBD). PBMCs were activated and expanded with human CD3/CD28 T Cell activator (25 µg/mL, Stemcell) and IL-2 (20 ng/mL, MCE). Activated PBMCs were then co-cultured with breast cancer cells at an effector to target (E: T) ratio of 10:1 for 72 h. The procedures were carried out as previously described [11, 19, 20]. After co-culture, apoptosis and cytokine release were analyzed by flow cytometry.

### ELISA

To evaluate cytokine secretion, the supernatants were collected by centrifugation after the co-culture of tumor cells and PBMCs. The concentrations of human interferon-γ (IFN-γ) (Yuanjubio) and granzyme B (GzmB) (Jonlnbio) were quantified using specific ELISA kits according to the manufacturers’ instructions.

### Animal model

Female BALB/c mice (6–8 weeks old) were housed under specific pathogen-free (SPF) conditions with a 12 h light/dark cycle, 50% humidity, and 25 °C temperature. After acclimation, mice were randomly assigned to two groups and received subcutaneous injection of 5 × 10^5^ shCtrl or shPSMD14 4T1 cells. Once tumors formed, body weight and tumor size were measured every three days, with volume calculated as: volume = (length × width^2^) / 2. When tumor volumes reached 80–100 mm^3^, each group was further randomized to receive either PBS or anti-PD-1 treatment (*n* = 5 per group). Mice were treated with PBS or anti-PD-1 therapy (100 µg per mouse, Abinvivo) every three days. At the endpoint, mice were euthanized, and tumor tissues were excised and weighed, then digested for analysis of tumor-infiltrating immune cells by flow cytometry. All animal procedures were approved by the Animal Ethics Committee of Harbin Medical University and were conducted in accordance with the ARRIVE guidelines.

### Flow cytometry and isolation of tumor infiltrating immune cells

The procedure was performed as previously described [11, 21]. Briefly, tumor tissues from each group were minced and digested in freshly prepared solution for 30 min at 37 °C (Collagenase IV, 3 mg/mL + DNase I, 0.2 mg/mL, Solarbio). After digestion, red blood cells were lysed for 3–5 min. Cell suspensions were then blocked with anti-CD16/CD32 antibodies and stained with fluorochrome-conjugated antibodies against surface markers, along with Zombie NIR viability dye (BioLegend). For intracellular and nuclear cytokine staining, cells were stimulated with a Cell Stimulation and Protein Transport Inhibitor Kit and processed with an intracellular or nuclear fixation permeabilization kit. Stained cells were analyzed using a flow cytometer. Data were analyzed using FlowJo software (version 10.10.0). Detailed antibody information is provided in Table S4.

### Bioinformatics analysis

The Cancer Genome Atlas (TCGA) database was used to evaluate PSMD14 expression and its correlation with immune checkpoint molecules (http://portal.gdc.cancer.gov). Immune cell infiltration was evaluated using ssGSEA. Survival analysis was performed via the Kaplan-Meier Plotter database (https://kmplot.com/analysis/). PSMD14 expression across different breast cancer subtypes was examined using the UALCAN database (https://ualcan.path.uab.edu/). The GSE177043 dataset was downloaded from the Gene Expression Omnibus (GEO) for analysis of immune-related phenotypes (https://www.ncbi.nlm.nih.gov/geo/).

### Statistical analysis

Statistical analysis was accomplished with GraphPad Prism software. All experiments were conducted independently at least three times. Comparisons between two groups were made using Student’s t-test, while comparisons among multiple groups were performed using one-way or two-way ANOVA. Data are presented as mean ± SD. Statistical significance was defined as follows: ns (not significant), *P < 0.05, **P < 0.01, ***P < 0.001, and ****P < 0.0001.

## Results

### PSMD14 is upregulated and associated with the tumor microenvironment in breast cancer

**Fig. 1:**
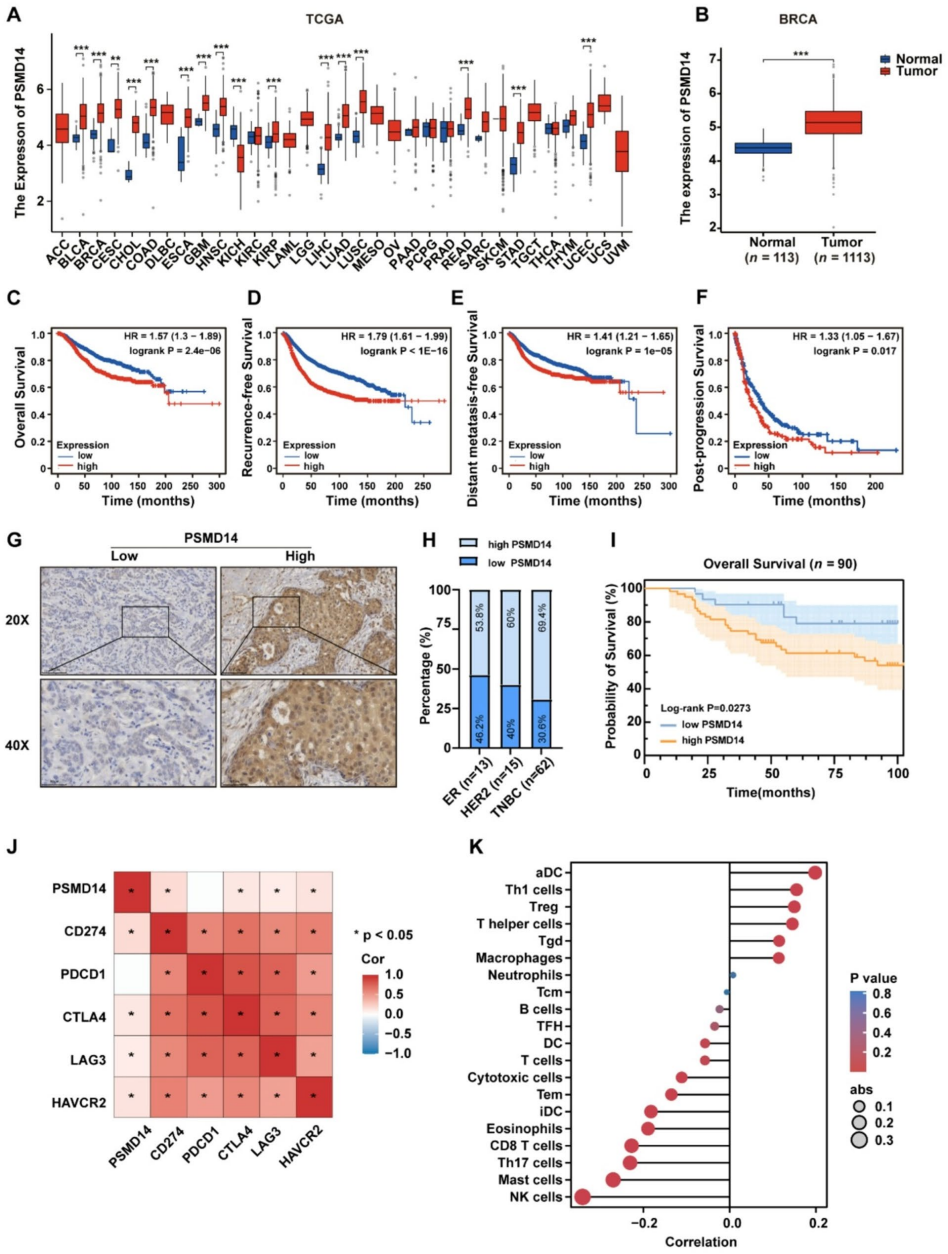
PSMD14 is upregulated and associated with the tumor microenvironment in breast cancer. **A** Pan-cancer analysis of PSMD14 expression in the TCGA database. **B** Comparative analysis of PSMD14 expression in normal tissues (*n =* 113) and breast cancer tissues (*n =* 1,113) in the TCGA database. **C-F** Survival analyses of OS, RFS, DMFS, and PPS according to PSMD14 expression in the TCGA-BRCA cohort. **G** Representative IHC staining of PSMD14 in breast cancer tissues (*n =* 90). Scale bars: 100 μm (upper panels) and 50 μm (lower panels). **H** Distribution of molecular subtypes between high and low PSMD14 expression. **I** OS analysis between high and low PSMD14 expression (*n =* 90). **J** Correlation of PSMD14 with immune checkpoint molecules. **K** Correlation between PSMD14 and tumor immune cell infiltration was analyzed by ssGSEA enrichment. Data are presented as mean ± SD. *P < 0.05, **P < 0.01, ***P < 0.001

To explore PSMD14 expression in breast cancer, we performed a pan-cancer analysis using TCGA data. The results showed that PSMD14 was upregulated in multiple cancers and significantly increased in breast cancer (Fig. 1A-B). Survival analysis using the Kaplan-Meier Plotter database indicated that PSMD14 was linked to poor clinical outcomes, including overall survival (OS), recurrence-free survival (RFS), distant metastasis-free survival (DMFS), and post-progression survival (PPS) (Fig. 1C-F). Subtype analysis based on the UALCAN database showed that PSMD14 was highest in TNBC, followed by HER2 and Luminal breast cancer (Fig. S1A).

To further investigate PSMD14 expression in breast cancer, we performed IHC on 90 breast cancer tissues (Table S1). The results showed that PSMD14 expression was markedly higher in TNBC compared with other subtypes (Fig. 1G-H). Elevated PSMD14 expression was also associated with poor OS (Fig. 1I). Among these patients, 34 had previously received immunotherapy (Table S2). Survival analysis showed that patients with low PSMD14 expression had longer OS (Fig. S1B). Moreover, patients with elevated PSMD14 expression exhibited reduced objective response rates (35% vs. 71.4%) (Fig. S1C).

Correlation analysis based on the TCGA-BRCA dataset showed that PSMD14 expression positively correlated with several immune checkpoint molecules, including CD274 (PD-L1), suggesting a potential role in immune regulation (Fig. 1J; Fig. S1D-G). Immune infiltration analysis using ssGSEA further indicated that PSMD14 was associated with multiple immune cell populations (Fig. 1K). Notably, PSMD14 expression was negatively correlated with CD8⁺ T cells and cytotoxic cells (Fig. S1H-I), but positively with regulatory T cells (Tregs) and macrophages (Fig. S1J-K). Analysis of the GSE177043 dataset further supported these findings, showing that elevated PSMD14 expression was associated with poorer prognosis (Fig. S1L-M). In addition, tumors with low PSMD14 expression were more likely to exhibit an immune-inflamed phenotype compared with controls (52.4% vs. 35%), whereas immune-desert tumors accounted for only 14.3%, markedly lower than in the PSMD14 high expression group (45%) (Fig. S1N). These findings suggest that low PSMD14 expression may be associated with improved response to immunotherapy in breast cancer patients.

### PSMD14 positively regulates PD-L1 and enhances its protein stability in breast cancer

**Fig. 2:**
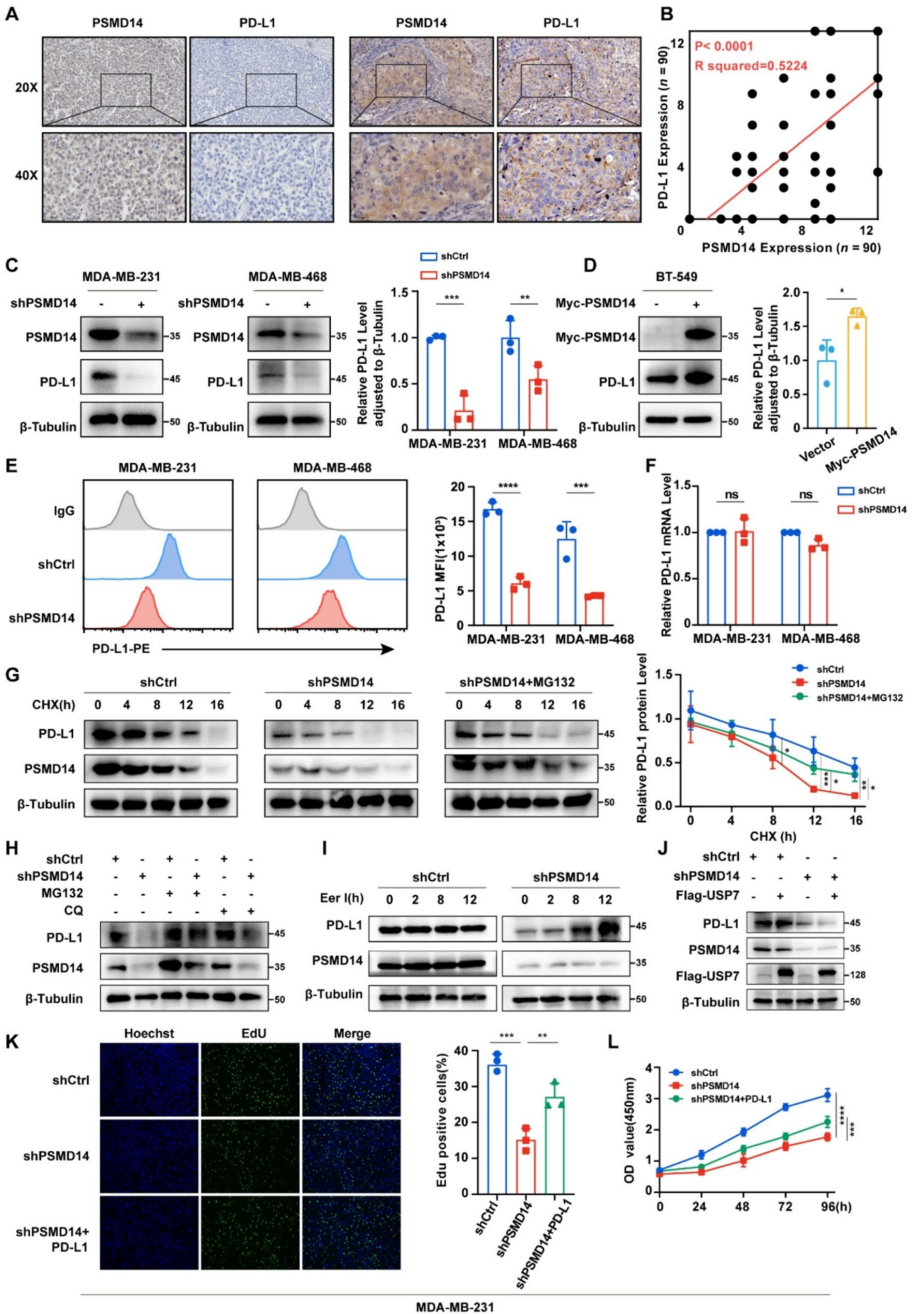
PSMD14 positively regulates PD-L1 and enhances its protein stability in breast cancer. **A** Representative IHC staining of PSMD14 and PD-L1 in breast cancer tissues (*n =* 90). Scale bars: 100 μm (upper panels) and 50 μm (lower panels). **B** Correlation analysis of IHC scores between PSMD14 and PD-L1. **C-D** Western blot analysis of PD-L1 and PSMD14 expression in MDA-MB-231 and MDA-MB-468 cells (**C**) and BT-549 cells (**D**). **E** Flow cytometry analysis of PD-L1 expression in breast cancer cells following PSMD14 knockdown, with quantification of mean fluorescence intensity (MFI). **F** qRT-PCR analysis of PD-L1 mRNA expression in breast cancer cells following PSMD14 knockdown. **G** CHX chase assay of PD-L1 protein half-life in MDA-MB-231 cells following PSMD14 knockdown in the presence or absence of MG132 (10 µM). **H** Western blot analysis of PD-L1 and PSMD14 expression in MDA-MB-231 cells treated with MG132 (10 µM) or CQ (20 µM). **I** Western blot analysis of PD-L1 expression in MDA-MB-231 cells at 0, 2, 8, and 12 h following Eer I (10 µM) treatment. **J** PD-L1 expression in shCtrl and shPSMD14 MDA-MB-231 cells following USP7 overexpression. **K-L** EdU and CCK-8 assays in MDA-MB-231 cells treated with shCtrl, shPSMD14, or shPSMD14 + PD-L1. Data are presented as mean ± SD (*n =* 3). ns, not significant, *P < 0.05, **P < 0.01, ***P < 0.001, ****P < 0.0001

To investigate the role of PSMD14 in breast cancer, stable breast cancer cell lines with PSMD14 knockdown or overexpression were established (Fig. S2A-C). Building on our previous findings [22], knockdown of PSMD14 inhibited breast cancer cell proliferation (Fig. S2D-G), whereas PSMD14 overexpression promoted cell growth (Fig. S2H-I). Further analysis showed a positive correlation between PSMD14 and PD-L1 in breast cancer tissues (*R* = 0.5224, *P* < 0.0001, *n =* 90) (Fig. 2A-B). Similar results were observed in cell lines, where PSMD14 knockdown reduced PD-L1 levels (Fig. 2C), whereas PSMD14 overexpression increased PD-L1 expression (Fig. 2D). Given that PD-L1 primarily acts at the cell membrane, we performed flow cytometry and confirmed that PSMD14 knockdown decreased PD-L1 surface expression (Fig. 2E). In contrast, qRT-PCR analysis demonstrated that PSMD14 did not affect PD-L1 mRNA levels in MDA-MB-231 or MDA-MB-468 cells (Fig. 2F). These findings suggest that PSMD14 may regulate PD-L1 through PTM in breast cancer.

To test this hypothesis, we assessed protein stability using CHX chase assays. PSMD14 knockdown significantly shortened the half-life of PD-L1 protein, and this degradation was largely reversed by the proteasome inhibitor MG132 (Fig. 2G). Conversely, PSMD14 overexpression prolonged PD-L1 stability (Fig. S3A). The ubiquitin-proteasome system (UPS) and the autophagy-lysosome pathway are two major routes for protein degradation. We discovered that MG132 restored PD-L1 levels, whereas chloroquine (CQ) had no effect in breast cancer cells, indicating that PD-L1 is primarily degraded through the UPS (Fig. 2H; Fig. S3B). UPS-mediated degradation of PD-L1 is dependent on the endoplasmic reticulum-associated degradation (ERAD) pathway. Treatment with the ERAD inhibitor Eeyarestatin I (Eer I) resulted in time-dependent accumulation of PD-L1 protein (Fig. 2I; Fig. S3C). Together, these results suggest that PSMD14 regulates PD-L1 stability through the UPS pathway in breast cancer.

To distinguish the role of PSMD14 from other DUBs, we compared its function with USP7 and CSN5, both reported to stabilize PD-L1 through deubiquitination. Overexpression of USP7 or CSN5 markedly increased PD-L1 levels; however, this effect was absent upon PSMD14 knockdown (Fig. 2J; Fig. S4A-C). Consistently, CHX chase assays showed that USP7 or CSN5 failed to extend the half-life of PD-L1 in PSMD14-knockdown cells (Fig. S4D-E). Notably, PSMD14 depletion had no effect on the abundance or stability of USP7 and CSN5. These findings indicate that PSMD14 regulates PD-L1 independently of USP7 or CSN5, playing a non-redundant role. Moreover, PD-L1 overexpression markedly enhanced cell proliferation in PSMD14-knockdown cells (Fig. 2K-L). Conversely, PD-L1 knockdown attenuated the proliferative effect induced by PSMD14 overexpression (Fig. S4F-G). Together, these results demonstrate that PSMD14 promotes breast cancer cell proliferation by upregulating PD-L1.

### PSMD14 interacts with the intracellular domain of PD-L1 in breast cancer

**Fig. 3:**
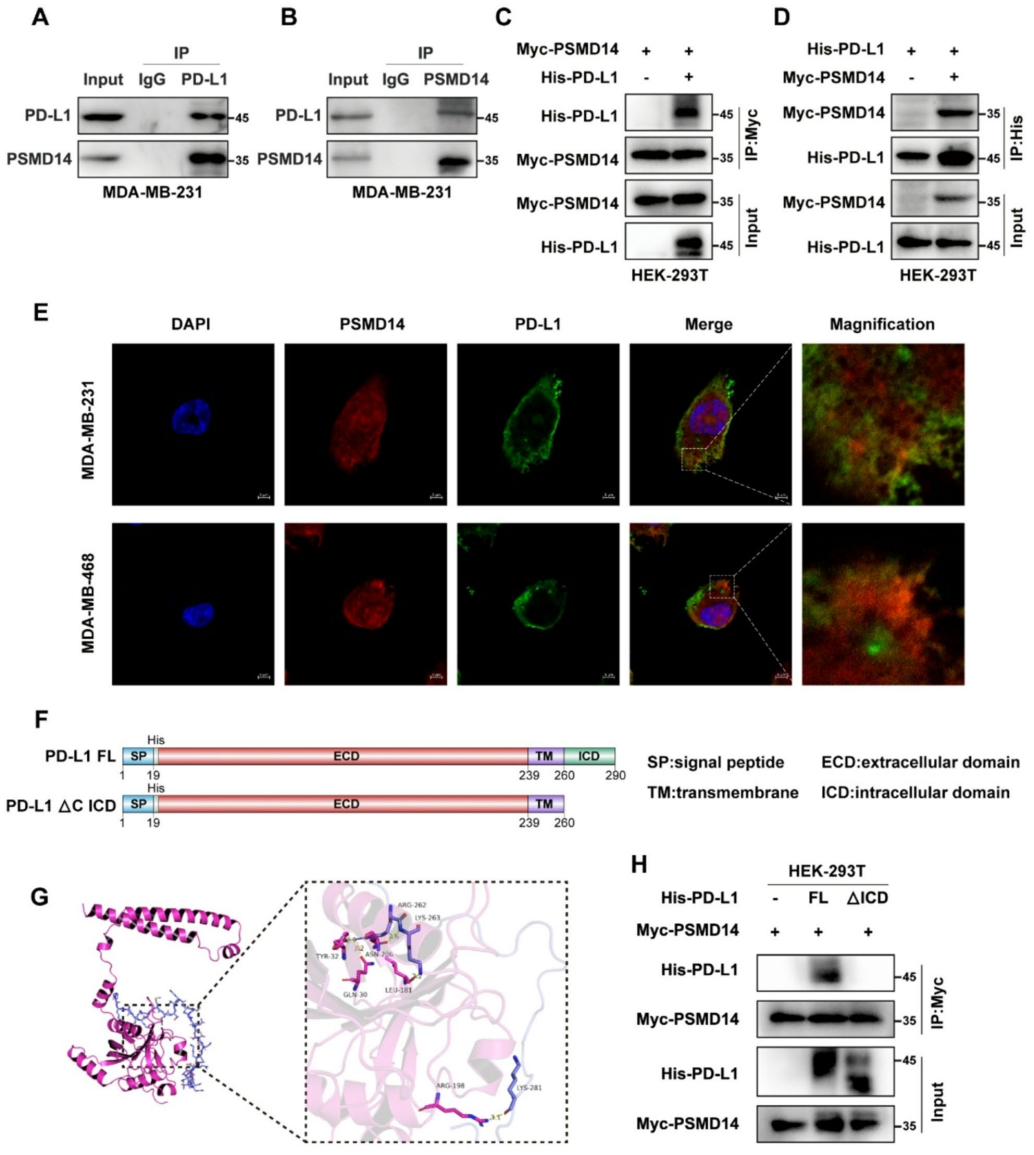
PSMD14 interacts with the intracellular domain of PD-L1 in breast cancer. **A-B** Endogenous Co-IP analysis of PSMD14 and PD-L1 in MDA-MB-231 cells. **C-D** Exogenous Co-IP analysis of Myc-PSMD14 and His-PD-L1. Immunoprecipitates were analyzed by western blot using anti-Myc (**C**) and anti-His (**D**) antibodies. **E** IF analysis of PSMD14 and PD-L1 co-localization in MDA-MB-231 and MDA-MB-468 cells. **F** Schematic representation of full-length PD-L1 and ΔICD plasmids. **G** Molecular docking model predicting the binding sites between PSMD14 and PD-L1 ICD. **H** Co-IP assay detecting the interaction between Myc-PSMD14 and full-length PD-L1 or ΔICD truncation mutant

Having established a positive association between PSMD14 and PD-L1, we next explored the underlying regulatory mechanism in breast cancer. Endogenous Co-IP assays demonstrated that PSMD14 interacts with PD-L1 in breast cancer cells (Fig. 3A-B). This interaction was further validated by exogenous Co-IP in HEK-293T cells co-transfected with Myc-PSMD14 and His-PD-L1 (Fig. 3C-D). IF analysis showed that PSMD14 and PD-L1 co-localize in the cytoplasm (Fig. 3E), suggesting a potential functional interaction. As PD-L1 is a transmembrane protein consisting of a signal peptide (SP), extracellular domain (ECD), transmembrane region (TM), and intracellular domain (ICD) (Fig. 3F) [23], we next examined which region mediates this interaction. Molecular docking simulations predicted a potential binding between PSMD14 and the PD-L1 ICD (Fig. 3G). Co-IP assays using full-length (FL) PD-L1 and its intracellular domain deletion mutant (ΔICD) showed that deletion of the ICD abolished its interaction with PSMD14 (Fig. 3H). These findings indicate that PSMD14 binds to the ICD of PD-L1 and may regulate its stability through this interaction.

### PSMD14 stabilizes PD-L1 by reducing its K48-linked polyubiquitin chains

**Fig. 4:**
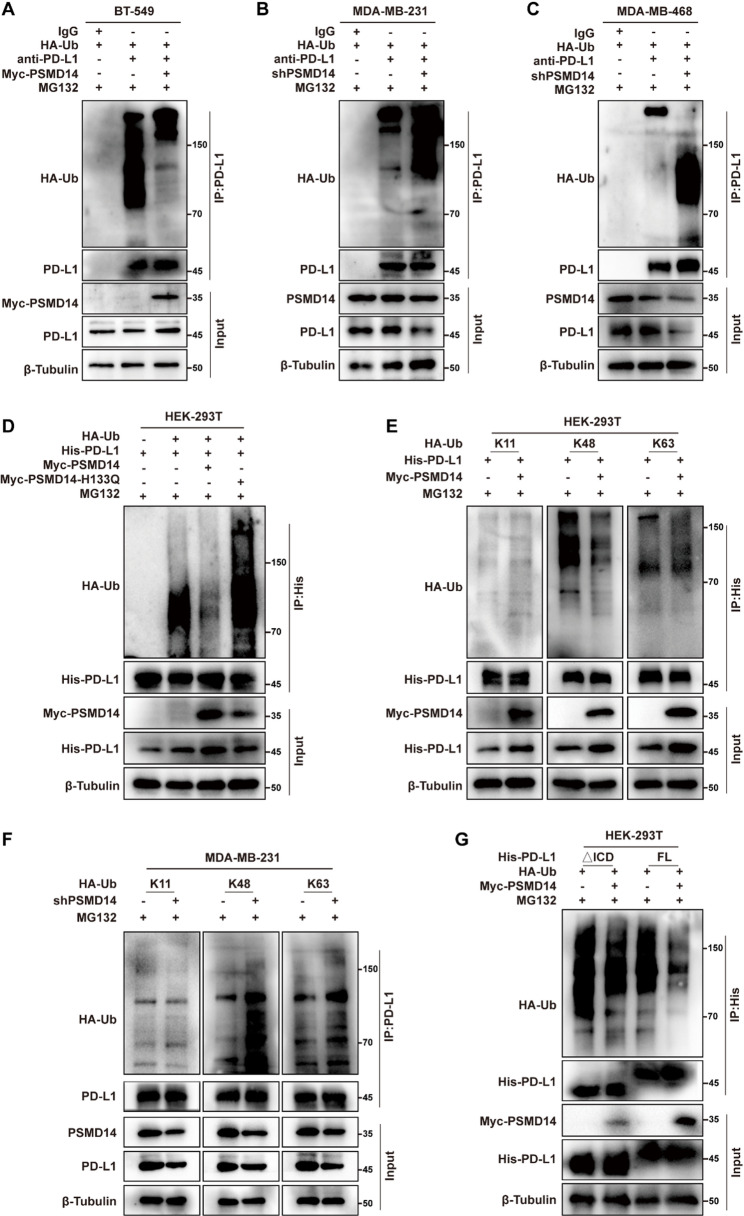
PSMD14 stabilizes PD-L1 by reducing its K48-linked polyubiquitin chains. **A-C** Western blot analysis of endogenous PD-L1 polyubiquitination in BT-549 (**A**), MDA-MB-231 (**B**), and MDA-MB-468 cells (**C**) following HA-Ub transfection. **D** Western blot analysis of PD-L1 polyubiquitination in HEK-293T cells co-transfected with Myc-PSMD14, Myc-PSMD14 H133Q, His-PD-L1, and HA-Ub. **E** Analysis of PD-L1 ubiquitin linkage types (K11, K48, and K63) in HEK-293T cells by western blot. **F** Analysis of PD-L1 ubiquitination with specific linkage types (K11, K48, and K63) in MDA-MB-231 cells following PSMD14 knockdown. **G** Western blot analysis of full-length PD-L1 and ΔICD polyubiquitination. All ubiquitination assays were performed following MG132 treatment

To determine whether PSMD14 regulates PD-L1 through deubiquitination, we performed ubiquitination assays following 6 h of MG132 treatment. In BT-549 cells, PSMD14 overexpression markedly reduced PD-L1 polyubiquitination (Fig. 4A; Fig. S5A), whereas PSMD14 knockdown increased PD-L1 ubiquitination in both MDA-MB-231 and MDA-MB-468 cells (Fig. 4B-C; Fig. S5B-C). To assess whether this effect depended on PSMD14 catalytic activity, HEK-293T cells were co-transfected with Myc-PSMD14, its catalytically inactive mutant (H133Q), and His-PD-L1 plasmids. Although Myc-PSMD14 reduced PD-L1 polyubiquitination, the H133Q mutant had no effect (Fig. 4D; Fig. S5D), indicating that the deubiquitinating activity of PSMD14 is required. Collectively, these findings indicate that PSMD14 is involved in the regulation of PD-L1 ubiquitination in a catalytic activity-dependent manner in breast cancer cells.

To explore the ubiquitin linkage types involved, we assessed its impact on K11-, K48-, and K63-linked polyubiquitination of PD-L1. In HEK-293T cells, we found that PSMD14 selectively reduced K48-linked polyubiquitin chains, with no significant effect on K11- or K63-linked chains (Fig. 4E; Fig. S5E). Consistently, PSMD14 inhibition led to a specific increase in K48-linked polyubiquitination of PD-L1 in MDA-MB-231 cells (Fig. 4F; Fig. S5F). Furthermore, based on our finding that PSMD14 interacts with the PD-L1 ICD (Fig. 3H), we assessed whether this interaction was required for deubiquitination. The results showed that PSMD14 reduced polyubiquitination of full-length PD-L1, whereas the ΔICD mutant was unaffected (Fig. 4G; Fig. S5G).

### PSMD14 inhibition enhances T cell-mediated tumor cell killing in breast cancer

**Fig. 5:**
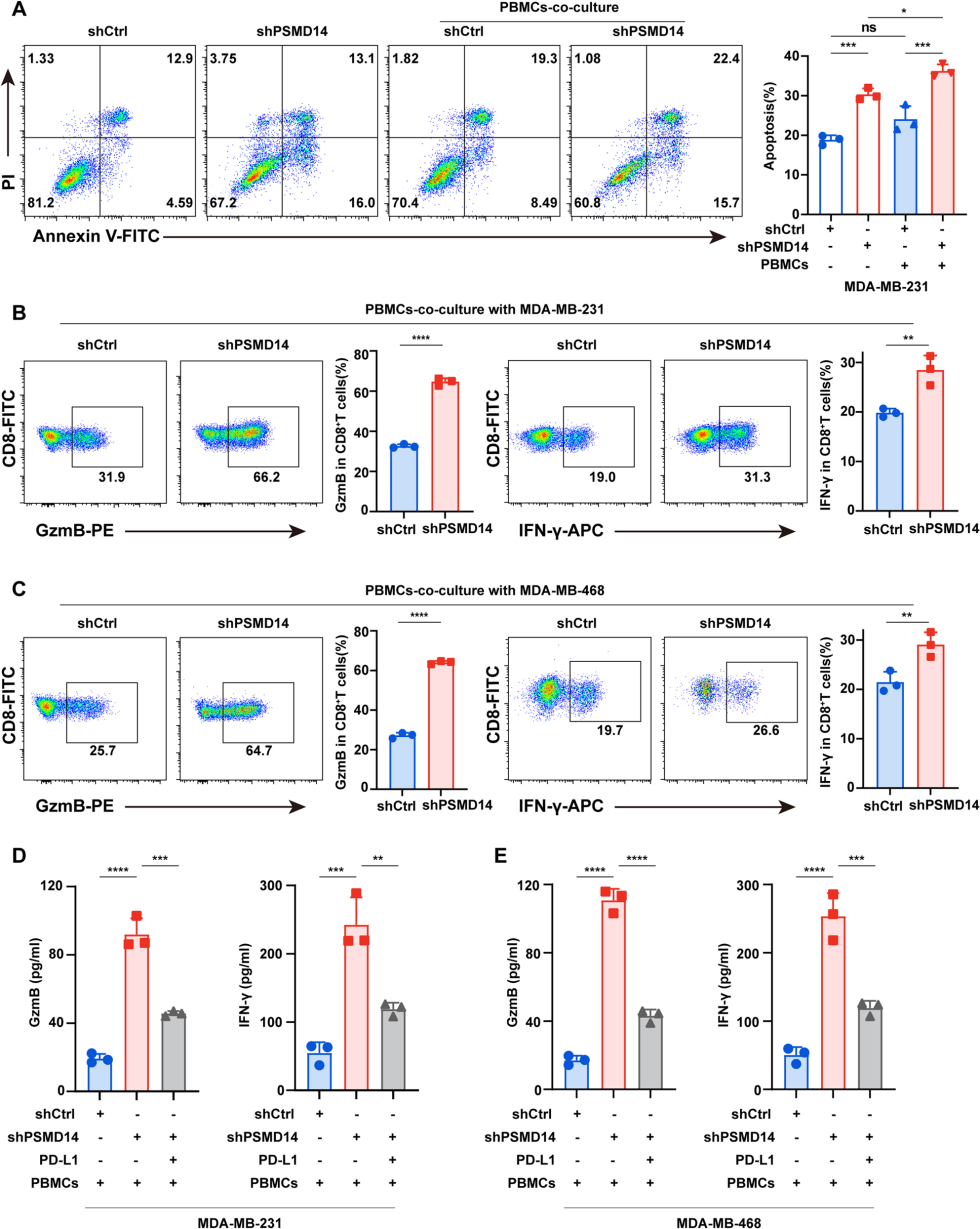
PSMD14 inhibition enhances T cell-mediated tumor cell killing in breast cancer. **A** Flow cytometric analysis of apoptosis in PSMD14-knockdown MDA-MB-231 cells following co-culture with PBMCs. Apoptosis was detected using Annexin V and propidium iodide (PI) double staining. **B-C** Flow cytometry analysis of intracellular GzmB and IFN-γ levels in CD8⁺ T cells after co-culture with PSMD14-knockdown MDA-MB-231 (**B**) and MDA-MB-468 (**C**) cells. **D-E** ELISA analysis of GzmB and IFN-γ levels in the supernatant after co-culture with MDA-MB-231 (**D**) and MDA-MB-468 (**E**) cells following PSMD14 knockdown with or without PD-L1 overexpression. Data are presented as mean ± SD (*n =* 3). ns, not significant, *P < 0.05, **P < 0.01, ***P < 0.001, ****P < 0.0001

PD-L1 binding to PD-1 inhibits T cell stimulation, leading to reduced secretion of cytotoxic factors and impaired tumor cell apoptosis. To determine whether PSMD14 influences T cell-mediated cytotoxicity in breast cancer, we performed co-culture assays with PBMCs. Flow cytometry revealed increased apoptosis in PSMD14-knockdown breast cancer cells, which was further enhanced following co-culture with PBMCs (Fig. 5A).

Given that T cell-mediated cytotoxicity is executed predominantly through the release of effector molecules such as GzmB and IFN-γ, we next measured their levels following co-culture. The levels of GzmB and IFN-γ, secreted upon co-culture with PBMCs, were significantly higher in PSMD14-knockdown MDA-MB-231 and MDA-MB-468 cells than in controls (Fig. 5B-C). To determine whether this effect depended on PD-L1, we overexpressed PD-L1 in PSMD14-knockdown cells. ELISA results showed that the increase in GzmB and IFN-γ secretion induced by PSMD14 inhibition was markedly attenuated upon PD-L1 overexpression (Fig. 5D-E). These findings indicate that PSMD14 modulates CD8⁺ T cell activation through PD-L1, thereby influencing the immune response in breast cancer.

### PSMD14 inhibition reshapes the tumor microenvironment and sensitizes breast cancer to anti-PD-1 therapy

**Fig. 6:**
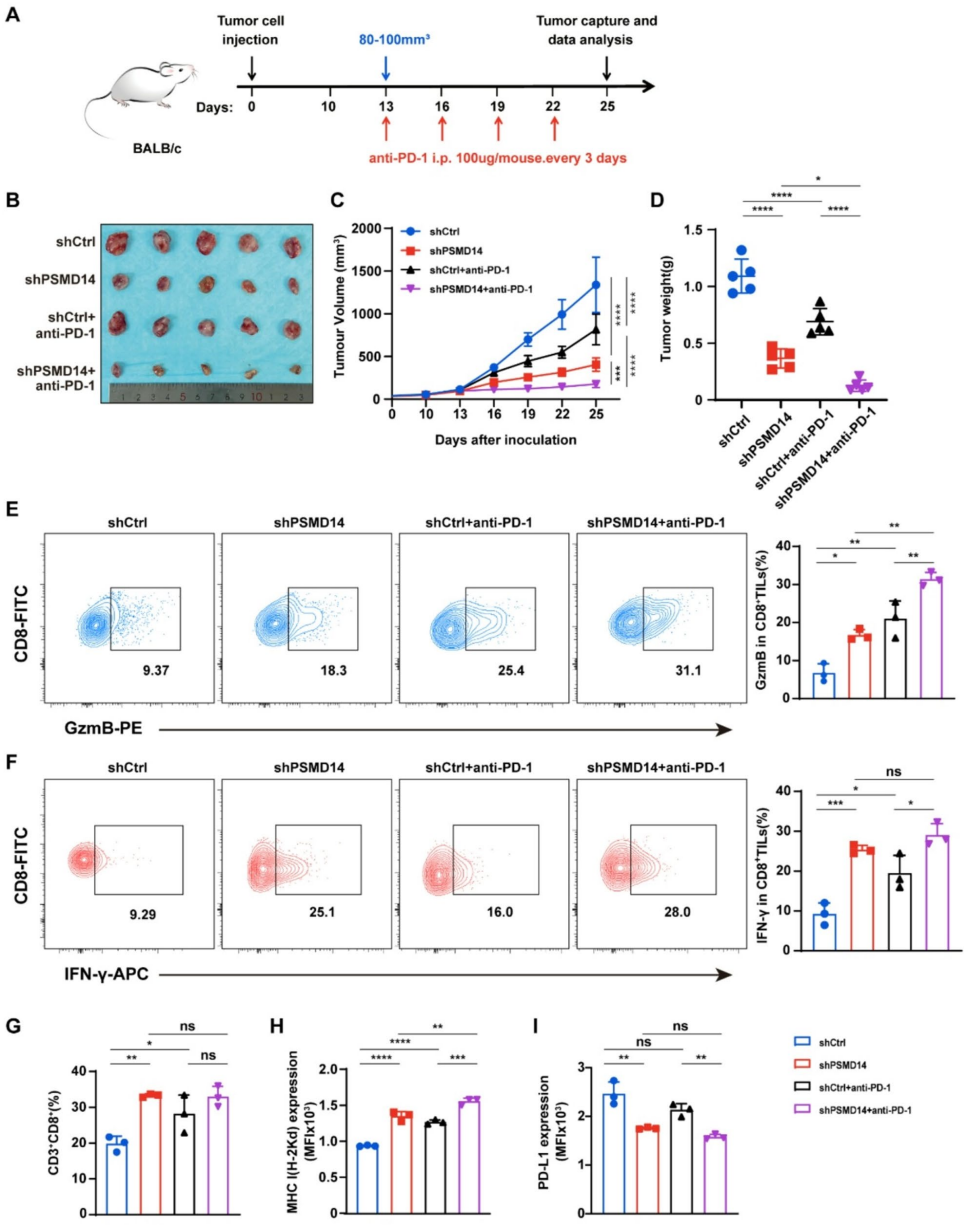
PSMD14 inhibition reshapes the tumor microenvironment and sensitizes breast cancer to anti-PD-1 therapy. **A** Schematic illustration of the in vivo experimental design and anti-PD-1 treatment schedule. **B** Photographs of tumors from each group in BALB/c mice (*n =* 5). **C-D** Tumor growth curves (**C**) and tumor weights (**D**) in BALB/c mice injected with 4T1 cells and treated with the indicated treatments (*n =* 5). **E-F** Flow cytometry analysis of the proportion of GzmB⁺ (**E**) and IFN-γ⁺ (**F**) tumor-infiltrating CD8⁺ T cells in each treatment group. **G** Flow cytometry analysis of the proportion of tumor-infiltrating CD8⁺ T cells. **H-I** Analysis of MHC I (**H**) and PD-L1 (**I**) expression by flow cytometry in each experimental group, with quantification of MFI. (E-I) *n =* 3 independent experiments. Data are presented as mean ± SD. ns, not significant, *P < 0.05, **P < 0.01, ***P < 0.001, ****P < 0.0001

To investigate the function of PSMD14 in vivo, we established a mouse xenograft model and administered anti-PD-1 treatment once the tumors reached 80–100 mm^3^ (Fig. 6A). Inhibition of PSMD14 greatly suppressed tumor growth, as evidenced by reductions in both tumor volume and weight. Combining shPSMD14 with anti-PD-1 treatment further enhanced the antitumor effect, producing a stronger response than either treatment alone (Fig. 6B-D). These findings indicate that PSMD14 promotes breast cancer growth in vivo and may influence tumor responsiveness to anti-PD-1 immunotherapy.

Flow cytometric analysis showed that PSMD14 inhibition increased the proportions of GzmB⁺ and IFN-γ⁺ CD8⁺ T cells within tumor tissues. This effect was further enhanced when combined with anti-PD-1 treatment, indicating a stronger activation of cytotoxic T cell responses (Fig. 6E-F). Consistently, CD8⁺ T cell infiltration was significantly elevated in the shPSMD14 group (Fig. 6G). Since T cell activation and function depend on antigen presentation, we found that PSMD14 knockdown increased major histocompatibility complex class I (MHC I) expression, which was further upregulated following anti-PD-1 treatment (Fig. 6H). In line with our earlier findings, PSMD14 depletion reduced PD-L1 expression, and combination therapy more effectively suppressed the PD-1/PD-L1 signaling axis (Fig. 6I). Together, these results reveal that PSMD14 inhibition enhances antitumor immunity and improves responsiveness to anti-PD-1 therapy.

**Fig. 7:**
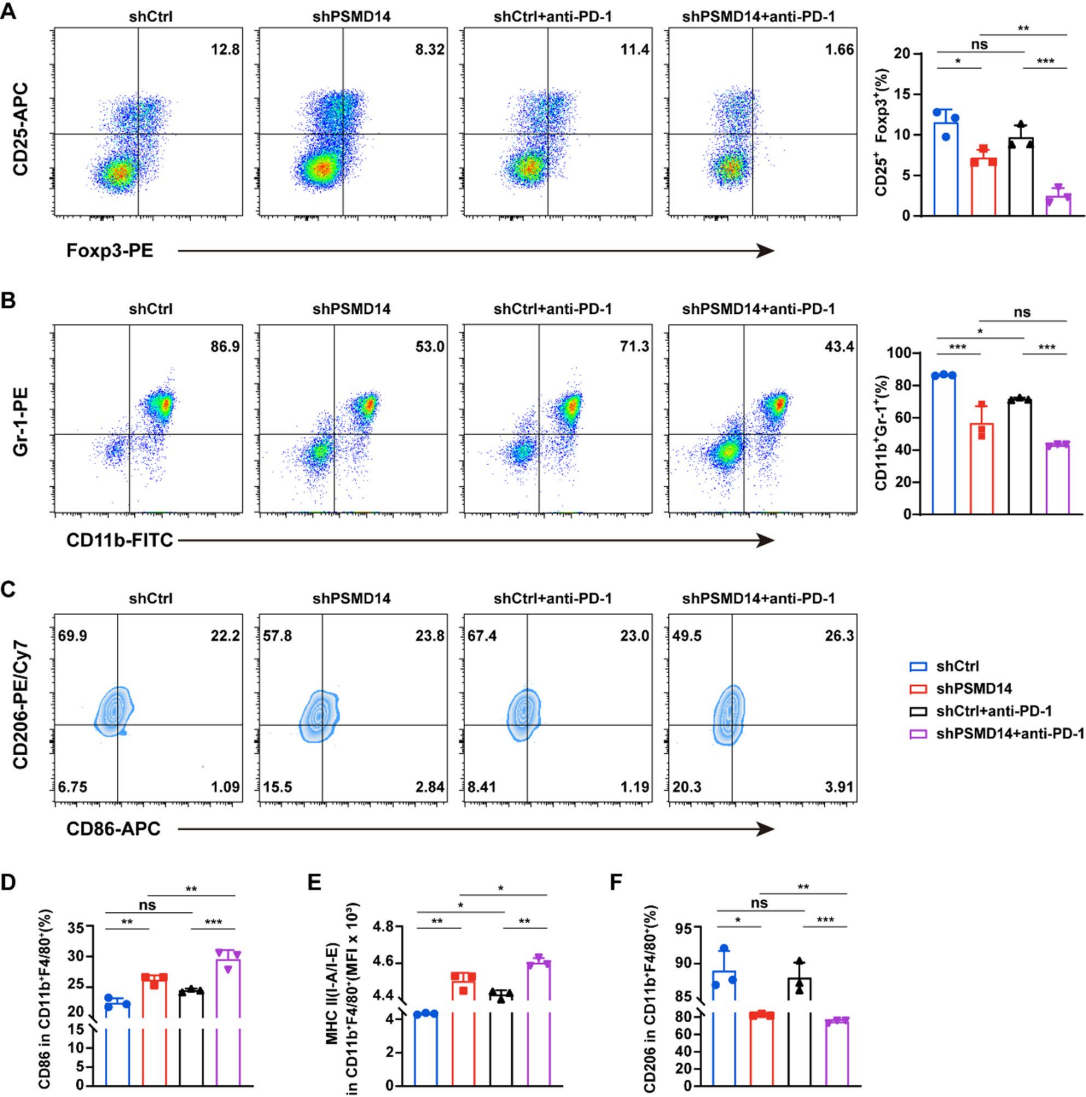
PSMD14 inhibition reshapes the tumor microenvironment and sensitizes breast cancer to anti-PD-1 therapy. **A** Flow cytometric analysis of CD25⁺Foxp3⁺ Tregs in tumor tissues from each group. **B** Flow cytometric analysis of CD11b⁺Gr-1⁺ MDSCs in tumor tissues from each group. **C** Flow cytometric analysis of the effect of PSMD14 on tumor-infiltrating macrophages. **D-E** Analysis of CD86 (**D**) and MHC II (**E**) expression in CD11b⁺F4/80⁺ M1 macrophages by flow cytometry. **F** Analysis of CD206 expression in CD11b⁺F4/80⁺ M2 macrophages by flow cytometry. Data are presented as mean ± SD (*n =* 3). ns, not significant, *P < 0.05, **P < 0.01, ***P < 0.001

In addition to CD8⁺ T cells, immunosuppressive cell populations such as Tregs and myeloid-derived suppressor cells (MDSCs) are present in the TME. Immune infiltration analysis showed that PSMD14 inhibition reduced the accumulation of CD25⁺Foxp3⁺ Tregs and CD11b⁺Gr-1⁺ MDSCs. Combination with anti-PD-1 therapy further decreased these populations, effectively alleviating the immunosuppressive TME in breast cancer (Fig. 7A-B). Tumor-associated macrophages (TAMs) are another key component of the TME, categorized into classically activated (M1) and alternatively activated (M2) macrophages. We therefore examined the impact of PSMD14 on TAMs (Fig. 7C). PSMD14 knockdown increased the expression of M1 macrophage markers, including CD86 and major histocompatibility complex class II (MHC II), while reducing the M2 marker CD206. This M1 polarization was further promoted by anti-PD-1 treatment (Fig. 7D-E), whereas the proportion of M2 macrophages continued to decline (Fig. 7F). Taken together, these results show that PSMD14 inhibition reshapes the TME by enhancing cytotoxic T cell activity, reducing immunosuppressive cell populations, and promoting M1-like macrophage polarization, ultimately strengthening antitumor immune responses in breast cancer.

## Discussion

**Fig. 8:**
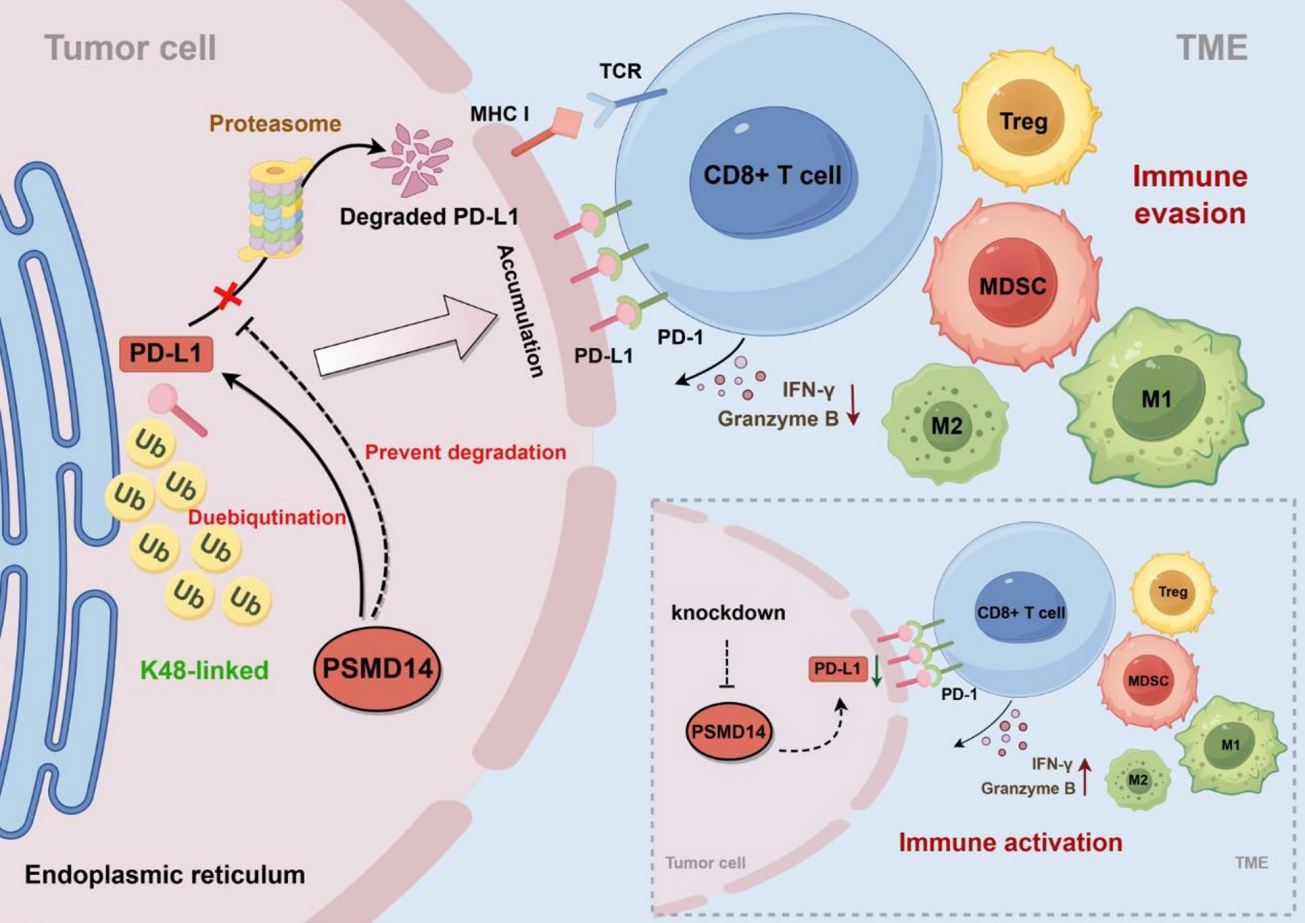
Schematic model illustrating the role of PSMD14 in regulating PD-L1 stability and the TME in breast cancer. Targeting PSMD14 promotes PD-L1 degradation by increasing K48-linked polyubiquitination and reshaping the TME, thereby sensitizing breast cancer to anti-PD-1 therapy

This study highlights the critical role of PSMD14 in regulating the immune response in breast cancer. Given its established oncogenic function in various tumors [24, 25], we found that PSMD14 is overexpressed and associated with poor prognosis in breast cancer. Using clinical samples and cell lines, we observed that PSMD14 expression positively correlated with PD-L1. Mechanistically, PSMD14 interacted with PD-L1 ICD and stabilized it by reducing K48-linked polyubiquitin chains. Inhibition of PSMD14 reduced PD-L1 expression, thereby promoting CD8⁺ T cell activation and increasing antitumor cytotoxicity. In addition, PSMD14 inhibition reshaped the TME by reducing immunosuppressive cell populations and modulating macrophage polarization. These findings suggest that PSMD14 represents an attractive therapeutic target to enhance the efficacy of immunotherapy in breast cancer (Fig. 8).

PD-L1 plays a pivotal role in tumor immune escape. Its interaction with PD-1 triggers phosphorylation of immunoreceptor tyrosine-based inhibitory and switch motifs, which suppresses CD28 signaling, impairs T cell function, and ultimately dampens antitumor immunity [26, 27]. PD-L1 expression is regulated at multiple levels, including transcriptional, post-transcriptional, and post-translational modifications [28–30]. Among these, ubiquitination is a key mechanism governing PD-L1 stability. For example, USP7 has been shown to stabilize PD-L1 by removing K48-linked ubiquitin chains, thereby promoting gastric cancer progression [31]. In this study, we observed a positive correlation between PSMD14 and PD-L1 at the protein level but not at the mRNA level, suggesting that PSMD14 regulates PD-L1 through PTMs. Protein homeostasis is maintained primarily through the UPS and the autophagy-lysosome pathway [32]. The ERAD pathway mediates the degradation of misfolded or unassembled proteins in the endoplasmic reticulum via retrotranslocation to the cytoplasm for proteasomal degradation. Previous studies have reported that USP2 and OTUB1 regulate PD-L1 stability through ERAD [11, 19]. Consistently, our data indicated that PSMD14 regulates PD-L1 stability mainly through the UPS, particularly via ERAD, thereby preventing its proteasomal degradation. Importantly, we confirmed the interaction between PSMD14 and the ICD of PD-L1 in breast cancer cells, providing a spatial basis for this regulatory mechanism and highlighting the PD-L1 ICD as the key region required for PSMD14-mediated stabilization.

Unlike other DUBs that function in response to inflammatory signals or specific stimuli, PSMD14’s unique positioning places it at a critical checkpoint within the UPS, determining whether substrate proteins are committed to proteasomal degradation. Although USP7 and CSN5 have been reported to regulate PD-L1 stability [33, 34], our findings indicate that PSMD14 functions independently and non-redundantly in regulating PD-L1. Nonetheless, potential cooperation between PSMD14 and other DUBs cannot be excluded and requires further investigation. Having established that PSMD14 regulates PD-L1 stability via the UPS, we found that PSMD14 modulated PD-L1 ubiquitination in a catalytic activity-dependent manner. This effect was dependent on the ICD of PD-L1, further supporting their interaction within the cytoplasm. Ubiquitin contains seven lysine residues (K6, K11, K27, K29, K33, K48, and K63) and one N-terminal methionine (M1) [35]. Among these, K48-linked ubiquitination primarily targets proteins for proteasomal degradation, whereas K63-linked ubiquitination participates in diverse activities, such as DNA damage repair [36, 37]. PSMD14 has been reported to stabilize SF3B4 by removing K63-linked ubiquitin chains in TNBC [15]. In our study, PSMD14 reduced PD-L1 K48-linked polyubiquitination, consistent with its role as a proteasome-associated DUB. Collectively, these findings indicate that PSMD14 prevents UPS-mediated degradation of PD-L1 by reducing its K48-linked ubiquitination in breast cancer.

Tumors are commonly classified into immune-inflamed, immune-excluded, and immune-desert phenotypes based on their TME [38]. Immune-inflamed tumors are characterized by abundant T cell infiltration within the tumor parenchyma and strong inflammatory signaling. Analysis of the GSE177043 dataset revealed that tumors with low PSMD14 expression had a higher proportion of the immune-inflamed phenotypes [39], suggesting that low PSMD14 expression is associated with a more immune-inflamed TME, which is typically more responsive to immunotherapy.

Immunotherapy response is influenced by PD-L1 expression, immune cell infiltration, and antigen presentation [40–42]. To date, the function of PSMD14 within the TME has been largely unexplored. Using both in vitro and in vivo models, we demonstrated that PSMD14 inhibition enhanced CD8⁺ T cell-mediated antitumor immunity. Specifically, PSMD14 inhibition promoted GzmB and IFN-γ release, key effectors of CD8⁺ T cell cytotoxicity. Antigen presentation is a critical step in T cell activation, where MHC I presents antigenic peptides to the T cell receptor, thereby mediating the recognition and activation of CD8⁺ T cells [43, 44]. Our data showed that PSMD14 knockdown increased MHC I expression, which was further enhanced by anti-PD-1 treatment, providing favorable conditions for sustained T cell activation. Tregs and MDSCs are major components of the immunosuppressive TME. Tregs primarily inhibit T cell activation by secreting inhibitory molecules such as IL-10 [45, 46], while MDSCs suppress immune responses by producing factors like arginase-1 [47, 48]. It has been reported that the combined loss of USP21 and USP22 reduces Foxp3 accumulation in the TME and restores CD8⁺ T cell function [49]. Consistent with this, our results showed that PSMD14 knockdown decreased the accumulation of Tregs and MDSCs. Although we did not further investigate the effects of PSMD14 on inhibitory cytokine secretion, these findings suggest that PSMD14 contributes to maintaining an immunosuppressive microenvironment. Moreover, PSMD14 suppression influenced TAMs. M1-like macrophages primarily exert antitumor immune functions by secreting pro-inflammatory cytokines such as TNF-α, which inhibit tumor progression [50, 51], whereas M2-like macrophages generally promote tumor development by releasing suppressive factors and reducing pro-inflammatory responses [52, 53]. Our data showed that PSMD14 inhibition increased M1 marker expression while reducing M2 markers. Combined with the decreased presence of Tregs and MDSCs, these results indicate that PSMD14 inhibition broadly remodels the TME toward a more antitumor, immune-active state.

Despite these findings, several limitations should be acknowledged. First, although our experiments support PSMD14-mediated regulation of PD-L1, direct visualization of these dynamic interactions using live-cell imaging or proximity labeling remains necessary. Second, while we show that PSMD14 stabilizes PD-L1 by regulating its ubiquitination in breast cancer cells, this has not been validated by in vitro deubiquitination assays; future studies are required to confirm whether PSMD14 directly deubiquitinates PD-L1 or functions within a protein complex. Third, we show that PSMD14 regulates T cell activation through PD-L1 in co-culture systems. However, how PSMD14 modulates other immune populations, whether via PD-L1, altered cytokine secretion, or other indirect mechanisms, remains unclear and warrants further investigation. Finally, while PD-L1 combined positive score (CPS) is widely used to stratify patients for PD-1/PD-L1 inhibitors, its predictive value remains complex [54]. The KEYNOTE-522 trial showed that even patients with CPS < 1 could benefit from pembrolizumab, highlighting variability in therapeutic responses [55]. In our cohort, CPS was not significantly associated with PSMD14, which may reflect the limited sample size and lack of longitudinal assessment. Larger studies with dynamic CPS monitoring are therefore warranted to clarify this relationship.

The development of DUB inhibitors is advancing rapidly, and combination strategies with immunotherapy are actively being explored. USP7 inhibitor combined with anti-PD-1 therapy has been reported to reduce prostate tumor burden in mice [56]. Several PSMD14 inhibitors, including Thiolutin, Capzimin, and O-phenanthroline (OPA), have been reported [57]. However, the present study did not evaluate pharmacological inhibition of PSMD14 *in vivo.* Future work will focus on evaluating the therapeutic potential of PSMD14 inhibitors in animal models to validate their efficacy in combination immunotherapy for breast cancer, providing a foundation for clinical translation.

## Conclusion

In conclusion, our study uncovers a critical post-translational regulatory pathway in which PSMD14 maintains PD-L1 stability by removing K48-linked polyubiquitin chains in breast cancer. Targeting PSMD14 not only relieves PD-L1-mediated inhibition of CD8⁺ T cells but also reshapes the TME, enhancing antitumor immunity. These findings establish PSMD14 as a promising therapeutic target that can synergize with anti-PD-1 therapy to overcome immune evasion, providing a mechanistic foundation for advancing combination immunotherapy in breast cancer.

## Supplementary Information

Supplementary Material 1: Fig. S1 PSMD14 is upregulated and associated with the tumor microenvironment in breast cancer. A Analysis of PSMD14 expression among breast cancer subtypes in the UALCAN database. B OS analysis evaluating the prognostic value of PSMD14 expression in breast cancer patients treated with PD-1/PD-L1 inhibitors (*n* = 34). C Correlation between PSMD14 expression and immunotherapy response in breast cancer. D-G Correlation analysis between PSMD14 and PD-L1, CTLA4, LAG3, HAVCR2 (Tim-3) in the TCGA-BRCA. H-K Correlation analysis between PSMD14 and enrichment of CD8⁺ T cells, cytotoxic cells, Tregs, and macrophages in the TCGA-BRCA. L-M OS and disease-free survival (DFS) analysis to evaluate the prognostic value of PSMD14 expression in the GSE177043 dataset. N Proportions of immune phenotypes by PSMD14 expression in the GSE177043 dataset. *P < 0.05. Fig. S2 PSMD14 promotes the proliferation of breast cancer cells. A-B Western blot analysis of PSMD14 protein levels following transfection with shPSMD14 in MDA-MB-231 (A) and MDA-MB-468 (B) cells. C PSMD14 protein levels following transfection with Myc-PSMD14 in BT-549 cells. D, F, H EdU assay for cell proliferation in MDA-MB-231 (D), MDA-MB-468 (F), and BT-549 (H) cells following PSMD14 knockdown or overexpression. E, G, I CCK-8 assay for cell proliferation in MDA-MB-231 (E), MDA-MB-468 (G), and BT-549 (I) cells following PSMD14 knockdown or overexpression. Data are presented as the mean ± SD (*n* = 3). *P < 0.05, **P < 0.01, ****P < 0.0001. Fig. S3 PSMD14 maintains PD-L1 stability through the UPS. A CHX chase assay of PD-L1 protein half-life in BT-549 cells following PSMD14 overexpression. B Western blot analysis of PD-L1 and PSMD14 expression in MDA-MB-468 cells treated with MG132 (10 µM) or CQ (20 µM). C Western blot analysis of PD-L1 expression in MDA-MB-468 cells at 0, 2, 8, and 12 h following Eer I (10 µM) treatment. Data are presented as mean ± SD (*n* = 3). *P < 0.05, **P < 0.01. Fig. S4 PSMD14-dependent maintenance of PD-L1 protein stability in breast cancer cells. A-B Western blot analysis of USP7 (A) and CSN5 (B) protein levels after transfection with Flag-USP7 or HA-CSN5 in MDA-MB-231 cells. C PD-L1 expression in shCtrl and shPSMD14 MDA-MB-231 cells following CSN5 overexpression. D-E CHX chase analysis of PD-L1 half-life following USP7 (D) or CSN5 (E) overexpression in MDA-MB-231 cells with shCtrl or shPSMD14. F-G EdU and CCK-8 assays in BT-549 cells treated with Vector, Myc-PSMD14, or Myc-PSMD14 + si-PD-L1. Data are presented as mean ± SD (*n* = 3). *P < 0.05, **P < 0.01, ***P < 0.001, ****P < 0.0001. Fig. S5 Quantitative analysis of ubiquitination assays. A-G Quantification of PD-L1 ubiquitination corresponding to Fig. 4. Ubiquitination levels were normalized to immunoprecipitated PD-L1. ns, not significant, *P < 0.05, **P < 0.01, ***P < 0.001. Table S1. Analysis of the correlation between PSMD14 level and clinical characteristics in breast cancer patients. Table S2. Analysis of the correlation between PSMD14 level and clinical characteristics in breast cancer patients treated with immunotherapy. Table S3. Targeting Sequence of shRNAs and siRNAs. Table S4. Antibodies and reagents. Table S5 Primer sequences used for qRT-PCR.

## Data Availability

The author can provide all the data used in this study upon reasonable request.

## Abbreviations

PD-1: Programmed death-1
PD-L1: Programmed death-ligand 1
TME: Tumor microenvironment
ICIs: Immune checkpoint inhibitors
PTM: Post-translational modification
DUBs: Deubiquitinating enzymes
TNBC: Triple-negative breast cancer
Tregs: Regulatory T cells
MDSCs: Myeloid-derived suppressor cells
siRNA: Small interfering RNA
MOI: Multiplicity of infection
IHC: Immunohistochemistry
TCGA: The Cancer Genome Atlas
CHX: Cycloheximide
Co-IP: Co-immunoprecipitation
IF: Immunofluorescence
PBMC: Peripheral blood mononuclear cell
OS: Overall survival
RFS: Recurrence-free survival
DMFS: Distant metastasis-free survival
PPS: Post-progression survival
CQ: Chloroquine
SP: Signal peptide
ECD: Extracellular domain
TM: Transmembrane
ICD: Intracellular domain
GzmB: Granzyme B
MHC I: Major histocompatibility complex class I
MHC II: Major histocompatibility complex class II
CPS: Combined positive score
ERAD: Endoplasmic reticulum associated degradation
OPA: O-phenanthroline
TAMs: Tumor-associated macrophages

## References

[1] Yi M, Niu M, Xu L, Luo S, Wu K. Regulation of PD-L1 expression in the tumor microenvironment. J Hematol Oncol. 2021;14(1):10. 33413496 10.1186/s13045-020-01027-5 PMC7792099

[2] Kim SB, Hwang S, Cha JY, Lee HJ. Programmed Death Ligand 1 Regulatory Crosstalk with Ubiquitination and Deubiquitination: Implications in Cancer Immunotherapy. Int J Mol Sci. 2024;25(5):2939. 10.3390/ijms25052939 PMC10932311 38474186

[3] Wu M, Huang Q, Xie Y, et al. Improvement of the anticancer efficacy of PD-1/PD-L1 blockade via combination therapy and PD-L1 regulation. J Hematol Oncol. 2022;15(1):24. 35279217 10.1186/s13045-022-01242-2 PMC8917703

[4] Popovic D, Vucic D, Dikic I. Ubiquitination in disease pathogenesis and treatment. Nat Med. 2014;20(11):1242–53. 25375928 10.1038/nm.3739

[5] Komander D, Rape M. The ubiquitin code. Annu Rev Biochem. 2012;81:203–29. 22524316 10.1146/annurev-biochem-060310-170328

[6] Sampson C, Wang Q, Otkur W, et al. The roles of E3 ubiquitin ligases in cancer progression and targeted therapy. Clin Transl Med. 2023;13(3):e1204. 36881608 10.1002/ctm2.1204 PMC9991012

[7] Cockram PE, Kist M, Prakash S, Chen SH, Wertz IE, Vucic D. Ubiquitination in the regulation of inflammatory cell death and cancer. Cell Death Differ. 2021;28(2):591–605. 33432113 10.1038/s41418-020-00708-5 PMC7798376

[8] Lange SM, Armstrong LA, Kulathu Y, Deubiquitinases. From mechanisms to their inhibition by small molecules. Mol Cell. 2022;82(1):15–29. 34813758 10.1016/j.molcel.2021.10.027

[9] Li Y, Reverter D. Molecular Mechanisms of DUBs Regulation in Signaling and Disease. Int J Mol Sci. 2021;22(3):986. 10.3390/ijms22030986 PMC7863924 33498168

[10] Torrado C, Ashton NW, D’Andrea AD, Yap TA. USP1 inhibition: A journey from target discovery to clinical translation. Pharmacol Ther. 2025;271:108865. 40274197 10.1016/j.pharmthera.2025.108865 PMC13411713

[11] Kuang Z, Liu X, Zhang N, et al. USP2 promotes tumor immune evasion via deubiquitination and stabilization of PD-L1. Cell Death Differ. 2023;30(10):2249–64. 37670038 10.1038/s41418-023-01219-9 PMC10589324

[12] He L, Yu C, Qin S et al. The proteasome component PSMD14 drives myelomagenesis through a histone deubiquitinase activity. Mol Cell. 2023;83(22):4000-16.e6. 10.1016/j.molcel.2023.10.019 37935198

[13] Jing C, Li X, Zhou M, et al. The PSMD14 inhibitor Thiolutin as a novel therapeutic approach for esophageal squamous cell carcinoma through facilitating SNAIL degradation. Theranostics. 2021;11(12):5847–62. 33897885 10.7150/thno.46109 PMC8058732

[14] Lu RS, Ren LK, Fei XB et al. PSMD14-Mediated LDHA Deubiquitination Upregulates ACLY Expression via H3K18 Lactylation to Promote Lipid Synthesis and Pancreatic Cancer Progression. Adv Sci (Weinh). 2025:e05762. 10.1002/advs.202505762 PMC12667490 41051446

[15] Yu Y, Hu J, Wang W, et al. Targeting PSMD14 combined with arachidonic acid induces synthetic lethality via FADS1 m(6)A modification in triple-negative breast cancer. Sci Adv. 2025;11(19):eadr3173. 40344056 10.1126/sciadv.adr3173 PMC12063657

[16] Yang P, Yang X, Wang D, et al. PSMD14 stabilizes estrogen signaling and facilitates breast cancer progression via deubiquitinating ERα. Oncogene. 2024;43(4):248–64. 38017133 10.1038/s41388-023-02905-1 PMC10798890

[17] Zhou M, Li X, Wang W, Wu J, Tan J. PSMD14/E2F1 Axis-Mediated CENPF Promotes the Metastasis of Triple-Negative Breast Cancer Through Inhibiting Ferroptosis. Cancer Sci. 2025;116(8):2281–95. 40365861 10.1111/cas.70064 PMC12317393

[18] Seymour L, Bogaerts J, Perrone A, et al. iRECIST: guidelines for response criteria for use in trials testing immunotherapeutics. Lancet Oncol. 2017;18(3):e143–52. 28271869 10.1016/S1470-2045(17)30074-8 PMC5648544

[19] Zhu D, Xu R, Huang X, et al. Deubiquitinating enzyme OTUB1 promotes cancer cell immunosuppression via preventing ER-associated degradation of immune checkpoint protein PD-L1. Cell Death Differ. 2021;28(6):1773–89. 33328570 10.1038/s41418-020-00700-z PMC8184985

[20] Jiao S, Xia W, Yamaguchi H, et al. PARP Inhibitor Upregulates PD-L1 Expression and Enhances Cancer-Associated Immunosuppression. Clin Cancer Res. 2017;23(14):3711–20. 28167507 10.1158/1078-0432.CCR-16-3215 PMC5511572

[21] Shi F, Li GJ, Liu Y, et al. USP19 deficiency enhances T-cell-mediated antitumor immunity by promoting PD-L1 degradation in colorectal cancer. Pharmacol Res. 2025;214:107668. 40020887 10.1016/j.phrs.2025.107668

[22] Liu Y, Wen S, Wang W, et al. PSMD14 promotes breast cancer progression by reducing K63-linked ubiquitination on FOXM1 and activating the PI3K/AKT/mTOR pathway. Int J Biol Macromol. 2025;327(Pt 1):147275. 40902741 10.1016/j.ijbiomac.2025.147275

[23] Cha JH, Chan LC, Li CW, Hsu JL, Hung MC. Mechanisms Controlling PD-L1 Expression in Cancer. Mol Cell. 2019;76(3):359–70. 31668929 10.1016/j.molcel.2019.09.030 PMC6981282

[24] Lv J, Zhang S, Wu H, et al. Deubiquitinase PSMD14 enhances hepatocellular carcinoma growth and metastasis by stabilizing GRB2. Cancer Lett. 2020;469:22–34. 31634528 10.1016/j.canlet.2019.10.025

[25] Lu J, Wu H, Zhan P, et al. PSMD14-mediated deubiquitination of CARM1 facilitates the proliferation and metastasis of hepatocellular carcinoma by inducing the transcriptional activation of FERMT1. Cell Death Dis. 2025;16(1):141. 10.1038/s41419-025-07416-3 PMC11868421 40016178

[26] Keir ME, Butte MJ, Freeman GJ, Sharpe AH. PD-1 and its ligands in tolerance and immunity. Annu Rev Immunol. 2008;26:677–704. 18173375 10.1146/annurev.immunol.26.021607.090331 PMC10637733

[27] Keir ME, Liang SC, Guleria I, et al. Tissue expression of PD-L1 mediates peripheral T cell tolerance. J Exp Med. 2006;203(4):883–95. 16606670 10.1084/jem.20051776 PMC2118286

[28] Messai Y, Gad S, Noman MZ, et al. Renal Cell Carcinoma Programmed Death-ligand 1, a New Direct Target of Hypoxia-inducible Factor-2 Alpha, is Regulated by von Hippel-Lindau Gene Mutation Status. Eur Urol. 2016;70(4):623–32. 26707870 10.1016/j.eururo.2015.11.029

[29] Miao Z, Li J, Wang Y, et al. Hsa_circ_0136666 stimulates gastric cancer progression and tumor immune escape by regulating the miR-375/PRKDC Axis and PD-L1 phosphorylation. Mol Cancer. 2023;22(1):205. 38093288 10.1186/s12943-023-01883-y PMC10718020

[30] Yang H, Zhang X, Lao M, et al. Targeting ubiquitin-specific protease 8 sensitizes anti-programmed death-ligand 1 immunotherapy of pancreatic cancer. Cell Death Differ. 2023;30(2):560–75. 36539510 10.1038/s41418-022-01102-z PMC9950432

[31] Wang Z, Kang W, Li O, et al. Abrogation of USP7 is an alternative strategy to downregulate PD-L1 and sensitize gastric cancer cells to T cells killing. Acta Pharm Sin B. 2021;11(3):694–707. 33777676 10.1016/j.apsb.2020.11.005 PMC7982505

[32] Krshnan L, van de Weijer ML, Carvalho P. Endoplasmic Reticulum-Associated Protein Degradation. Cold Spring Harb Perspect Biol. 2022;14(12):a041247. 10.1101/cshperspect.a041247 PMC9732900 35940909

[33] Lim SO, Li CW, Xia W, et al. Deubiquitination and Stabilization of PD-L1 by CSN5. Cancer Cell. 2016;30(6):925–39. 27866850 10.1016/j.ccell.2016.10.010 PMC5171205

[34] Yu ZZ, Liu YY, Zhu W et al. ANXA1-derived peptide for targeting PD-L1 degradation inhibits tumor immune evasion in multiple cancers. J Immunother Cancer. 2023;11(3):e006345. 10.1136/jitc-2022-006345 PMC10069584 37001908

[35] Martínez-Férriz A, Ferrando A, Fathinajafabadi A, Farràs R. Ubiquitin-mediated mechanisms of translational control. Semin Cell Dev Biol. 2022;132:146–54. 34952788 10.1016/j.semcdb.2021.12.009

[36] Xia T, Meng L, Xu G, Sun H, Chen H. TRIM33 promotes glycolysis through regulating P53 K48-linked ubiquitination to promote esophageal squamous cell carcinoma growth. Cell Death Dis. 2024;15(10):740. 39389957 10.1038/s41419-024-07137-z PMC11467421

[37] Tracz M, Bialek W. Beyond K48 and K63: non-canonical protein ubiquitination. Cell Mol Biol Lett. 2021;26(1):1. 33402098 10.1186/s11658-020-00245-6 PMC7786512

[38] Wu B, Zhang B, Li B, Wu H, Jiang M. Cold and hot tumors: from molecular mechanisms to targeted therapy. Signal Transduct Target Ther. 2024;9(1):274. 39420203 10.1038/s41392-024-01979-x PMC11491057

[39] Hammerl D, Martens JWM, Timmermans M, et al. Spatial immunophenotypes predict response to anti-PD1 treatment and capture distinct paths of T cell evasion in triple negative breast cancer. Nat Commun. 2021;12(1):5668. 34580291 10.1038/s41467-021-25962-0 PMC8476574

[40] Minnar CM, Chariou PL, Horn LA et al. Tumor-targeted interleukin-12 synergizes with entinostat to overcome PD-1/PD-L1 blockade-resistant tumors harboring MHC-I and APM deficiencies. J Immunother Cancer. 2022;10(6):e004561. 10.1136/jitc-2022-004561 PMC9240938 35764364

[41] Zhang Y, Zeng L, Wang M et al. RIG-I promotes immune evasion of colon cancer by modulating PD-L1 ubiquitination. J Immunother Cancer. 2023;11(9):e007313. 10.1136/jitc-2023-007313 PMC10537859 37758653

[42] Xiong W, Gao X, Zhang T, et al. USP8 inhibition reshapes an inflamed tumor microenvironment that potentiates the immunotherapy. Nat Commun. 2022;13(1):1700. 35361799 10.1038/s41467-022-29401-6 PMC8971425

[43] Rossjohn J, Gras S, Miles JJ, Turner SJ, Godfrey DI, McCluskey J. T cell antigen receptor recognition of antigen-presenting molecules. Annu Rev Immunol. 2015;33:169–200. 25493333 10.1146/annurev-immunol-032414-112334

[44] Rudolph MG, Stanfield RL, Wilson IA. How TCRs bind MHCs, peptides, and coreceptors. Annu Rev Immunol. 2006;24:419–66. 16551255 10.1146/annurev.immunol.23.021704.115658

[45] Plitas G, Konopacki C, Wu K, et al. Regulatory T Cells Exhibit Distinct Features in Human Breast Cancer. Immunity. 2016;45(5):1122–34. 27851913 10.1016/j.immuni.2016.10.032 PMC5134901

[46] Tay C, Tanaka A, Sakaguchi S. Tumor-infiltrating regulatory T cells as targets of cancer immunotherapy. Cancer Cell. 2023;41(3):450–65. 36917950 10.1016/j.ccell.2023.02.014

[47] He S, Zheng L, Qi C. Myeloid-derived suppressor cells (MDSCs) in the tumor microenvironment and their targeting in cancer therapy. Mol Cancer. 2025;24(1):5. 39780248 10.1186/s12943-024-02208-3 PMC11707952

[48] Zhang R, Dong M, Tu J, et al. PMN-MDSCs modulated by CCL20 from cancer cells promoted breast cancer cell stemness through CXCL2-CXCR2 pathway. Signal Transduct Target Ther. 2023;8(1):97. 36859354 10.1038/s41392-023-01337-3 PMC9977784

[49] Montauti E, Weinberg SE, Chu P, et al. A deubiquitination module essential for T(reg) fitness in the tumor microenvironment. Sci Adv. 2022;8(47):eabo4116. 36427305 10.1126/sciadv.abo4116 PMC9699683

[50] Ahirwar DK, Charan M, Mishra S, et al. Slit2 Inhibits Breast Cancer Metastasis by Activating M1-Like Phagocytic and Antifibrotic Macrophages. Cancer Res. 2021;81(20):5255–67. 34400395 10.1158/0008-5472.CAN-20-3909 PMC8631742

[51] Wu K, Yuan Y, Yu H, et al. The gut microbial metabolite trimethylamine N-oxide aggravates GVHD by inducing M1 macrophage polarization in mice. Blood. 2020;136(4):501–15. 32291445 10.1182/blood.2019003990 PMC7378459

[52] Genin M, Clement F, Fattaccioli A, Raes M, Michiels C. M1 and M2 macrophages derived from THP-1 cells differentially modulate the response of cancer cells to etoposide. BMC Cancer. 2015;15:577. 26253167 10.1186/s12885-015-1546-9 PMC4545815

[53] Shang L, Zhong Y, Yao Y, et al. Subverted macrophages in the triple-negative breast cancer ecosystem. Biomed Pharmacother. 2023;166:115414. 37660651 10.1016/j.biopha.2023.115414

[54] Fusco N, Ivanova M, Frascarelli C, et al. Advancing the PD-L1 CPS test in metastatic TNBC: Insights from pathologists and findings from a nationwide survey. Crit Rev Oncol Hematol. 2023;190:104103. 37595344 10.1016/j.critrevonc.2023.104103

[55] Schmid P, Cortes J, Pusztai L, et al. Pembrolizumab for Early Triple-Negative Breast Cancer. N Engl J Med. 2020;382(9):810–21. 32101663 10.1056/NEJMoa1910549

[56] Zhang J, Chen W, Zhang C, et al. Prostate Cancer Cells Secrete PD-1 in Exosomes to Enhance Myeloid-Derived Suppressor Cell Activity and Promote Tumor Immune Evasion. Cancer Res. 2025;85(18):3435–53. 40698651 10.1158/0008-5472.CAN-24-3748

[57] Song Y, Li S, Ray A, et al. Blockade of deubiquitylating enzyme Rpn11 triggers apoptosis in multiple myeloma cells and overcomes bortezomib resistance. Oncogene. 2017;36(40):5631–8. 28581522 10.1038/onc.2017.172 PMC5705032

