# Supplementary material for: Targeting PSMD14 enhances immunotherapy efficacy by promoting PD-L1 degradation and reshaping the tumor microenvironment in breast cancer
Source: J Exp Clin Cancer Res. 2026 Apr 14;45:124. doi: 10.1186/s13046-026-03710-w (PMC13188696; doi:10.1186/s13046-026-03710-w)
Supplement: Supplementary file 1 — Supplementary Material 1: Fig. S1 PSMD14 is upregulated and associated with the tumor microenvironment in breast cancer. A Analysis of PSMD14 expression among breast cancer subtypes in the UALCAN database. B OS analysis evaluating the prognostic value of PSMD14 expression in breast cancer patients treated with PD-1/PD-L1 inhibitors (n = 34). C Correlation between PSMD14 expression and immunotherapy response in breast cancer. D-G Correlation analysis between PSMD14 and PD-L1, CTLA4, LAG3, HAVCR2 (Tim-3) in the TCGA-BRCA. H-K Correlation analysis between PSMD14 and enrichment of CD8⁺ T cells, cytotoxic cells, Tregs, and macrophages in the TCGA-BRCA. L-M OS and disease-free survival (DFS) analysis to evaluate the prognostic value of PSMD14 expression in the GSE177043 dataset. N Proportions of immune phenotypes by PSMD14 expression in the GSE177043 dataset. *P < 0.05. Fig. S2 PSMD14 promotes the proliferation of breast cancer cells. A-B Western blot analysis of PSMD14 protein levels following transfection with shPSMD14 in MDA-MB-231 (A) and MDA-MB-468 (B) cells. C PSMD14 protein levels following transfection with Myc-PSMD14 in BT-549 cells. D, F, H EdU assay for cell proliferation in MDA-MB-231 (D), MDA-MB-468 (F), and BT-549 (H) cells following PSMD14 knockdown or overexpression. E, G, I CCK-8 assay for cell proliferation in MDA-MB-231 (E), MDA-MB-468 (G), and BT-549 (I) cells following PSMD14 knockdown or overexpression. Data are presented as the mean ± SD (n = 3). *P < 0.05, **P < 0.01, ****P < 0.0001. Fig. S3 PSMD14 maintains PD-L1 stability through the UPS. A CHX chase assay of PD-L1 protein half-life in BT-549 cells following PSMD14 overexpression. B Western blot analysis of PD-L1 and PSMD14 expression in MDA-MB-468 cells treated with MG132 (10 µM) or CQ (20 µM). C Western blot analysis of PD-L1 expression in MDA-MB-468 cells at 0, 2, 8, and 12 h following Eer I (10 µM) treatment. Data are presented as mean ± SD (n = 3). *P < 0.05, **P < 0.01. Fig. S4 PSMD14-depen [file 13046_2026_3710_MOESM1_ESM.docx]

**Supplemental Material**

**Targeting PSMD14 Enhances Immunotherapy Efficacy by Promoting PD-L1 Degradation and Reshaping the Tumor Microenvironment in Breast Cancer**

Shichao Wen^1,#^, Yuhan Liu^1,#^, Qi Liu^1^, Liqian Su^2^, Yuhua Wang^1^, Yiqiu Ma^1^, Jingxuan Wang^1,*^

1. Department of Medical Oncology, Harbin Medical University Cancer Hospital, Harbin, 150081, China.
2. Precision Medicine Center, Harbin Medical University Cancer Hospital, Harbin, 150081, China.

**^#^ Shichao Wen and Yuhan Liu contributed equally to this work.**

**^*^ Corresponding author: Jingxuan Wang (**wangjingxuan[@hrbmu.edu.cn](mailto:wangjingxuan@hrbmu.edu.cn)**)**

**Supplementary Figures**

**Supplementary Figure 1**


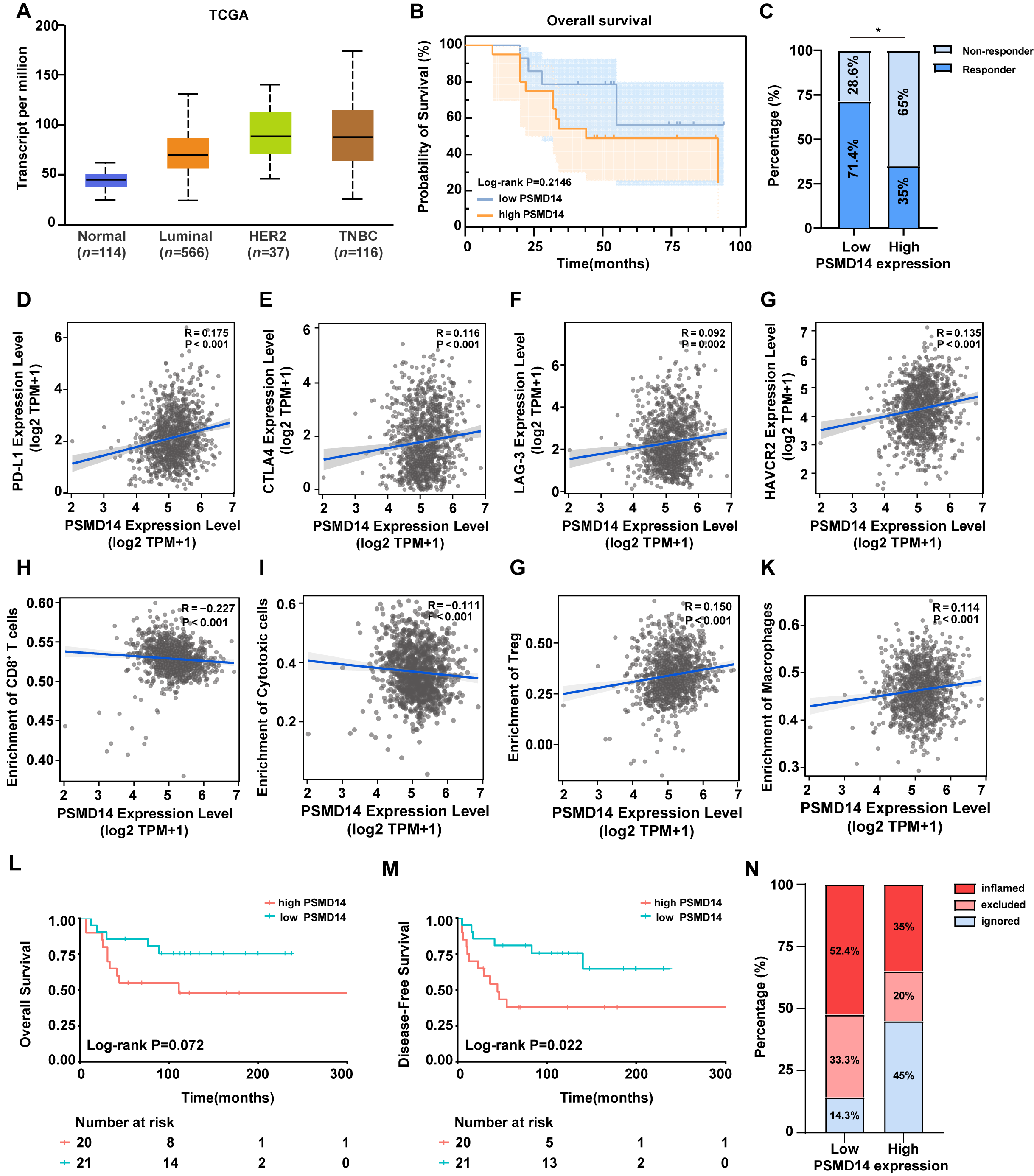


**Fig. S1 PSMD14 is upregulated and associated with the tumor microenvironment in breast cancer. A** Analysis of PSMD14 expression among breast cancer subtypes in the UALCAN database. **B** OS analysis evaluating the prognostic value of PSMD14 expression in breast cancer patients treated with PD-1/PD-L1 inhibitors (*n =* 34). **C** Correlation between PSMD14 expression and immunotherapy response in breast cancer. **D-G** Correlation analysis between PSMD14 and PD-L1, CTLA4, LAG3, HAVCR2 (Tim-3) in the TCGA-BRCA. **H-K** Correlation analysis between PSMD14 and enrichment of CD8⁺ T cells, cytotoxic cells, Tregs, and macrophages in the TCGA-BRCA. **L-M** OS and disease-free survival (DFS) analysis to evaluate the prognostic value of PSMD14 expression in the GSE177043 dataset. **N** Proportions of immune phenotypes by PSMD14 expression in the GSE177043 dataset. **P* < 0.05.

**Supplementary Figure 2**


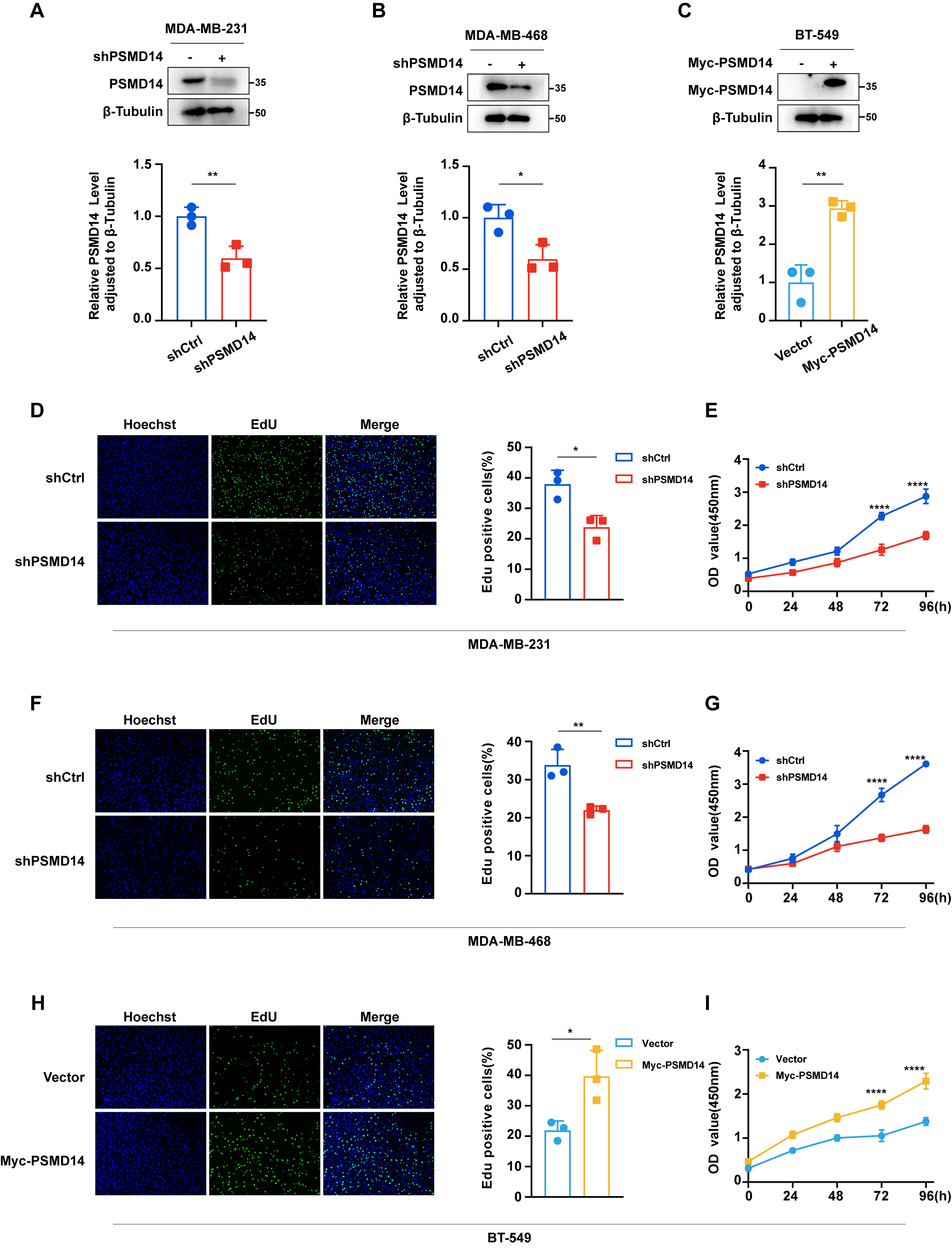


**Fig. S2 PSMD14 promotes the proliferation of breast cancer cells. A-B** Western blot analysis of PSMD14 protein levels following transfection with shPSMD14 in MDA-MB-231 (A) and MDA-MB-468 (B) cells. **C** PSMD14 protein levels following transfection with Myc-PSMD14 in BT-549 cells. **D, F, H** EdU assay for cell proliferation in MDA-MB-231 (D), MDA-MB-468 (F), and BT-549 (H) cells following PSMD14 knockdown or overexpression. **E, G, I** CCK-8 assay for cell proliferation in MDA-MB-231 (E), MDA-MB-468 (G), and BT-549 (I) cells following PSMD14 knockdown or overexpression. Data are presented as the mean ± SD (*n =* 3). **P* < 0.05, ***P* < 0.01, *****P* < 0.0001.

**Supplementary Figure 3**


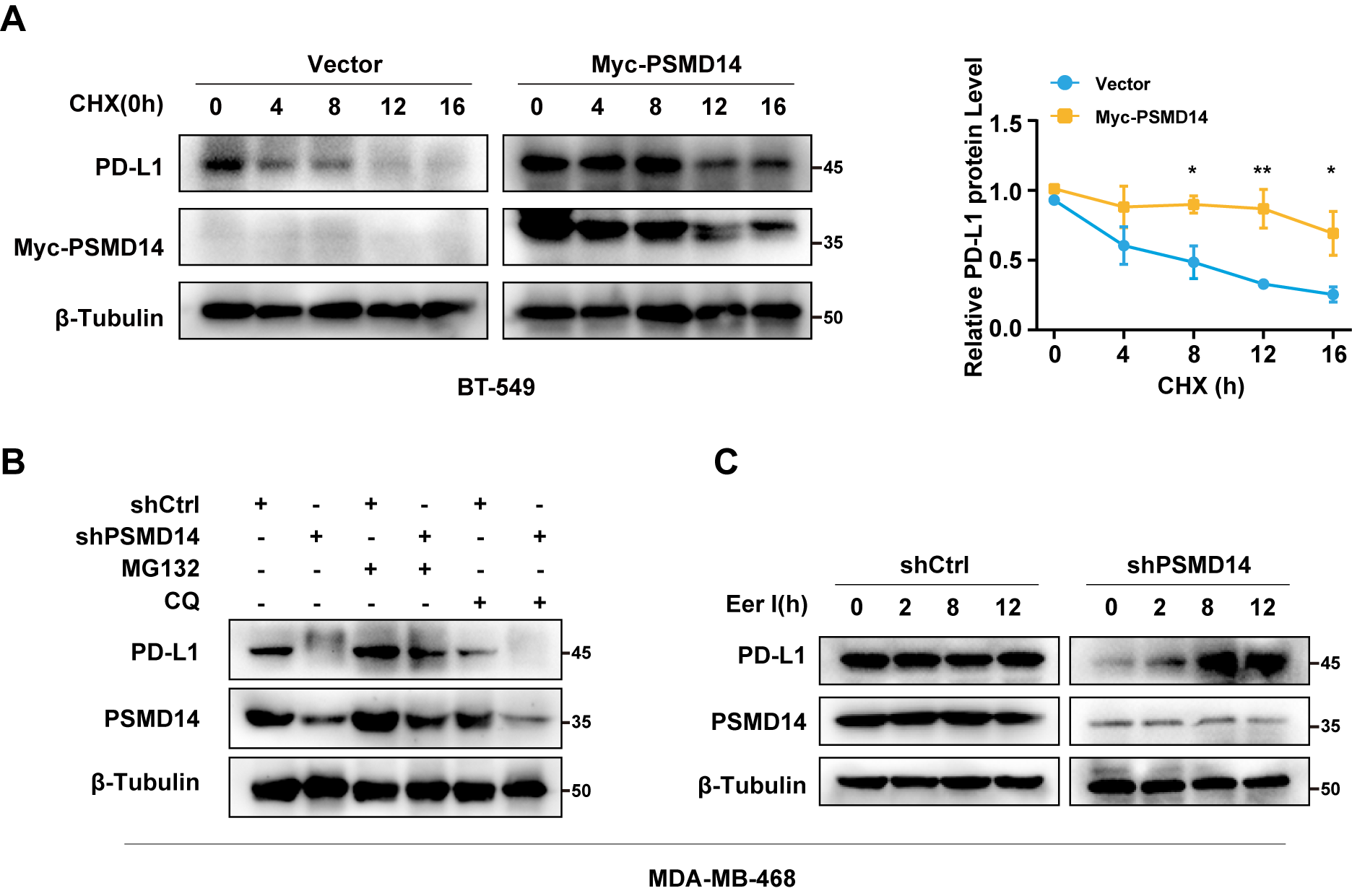


**Fig. S3 PSMD14 maintains PD-L1 stability through the UPS. A** CHX chase assay of PD-L1 protein half-life in BT-549 cells following PSMD14 overexpression. **B** Western blot analysis of PD-L1 and PSMD14 expression in MDA-MB-468 cells treated with MG132 (10 µM) or CQ (20 µM). **C** Western blot analysis of PD-L1 expression in MDA-MB-468 cells at 0, 2, 8, and 12 h following Eer I (10 µM) treatment. Data are presented as mean ± SD (*n =* 3). **P* < 0.05, ***P* < 0.01.

**Supplementary Figure 4**


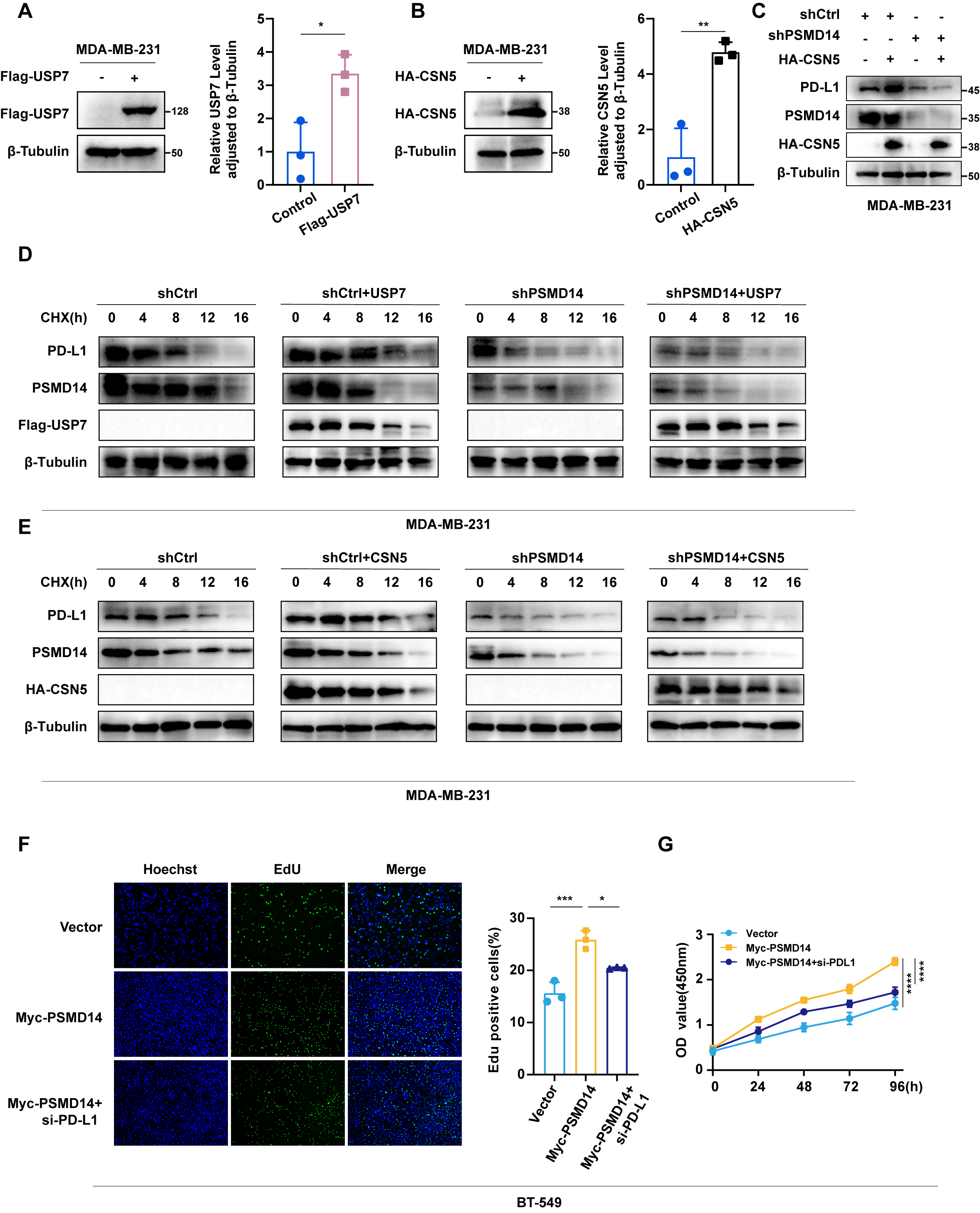


**Fig. S4 PSMD14-dependent maintenance of PD-L1 protein stability in breast cancer cells. A-B** Western blot analysis of USP7 (A) and CSN5 (B) protein levels after transfection with Flag-USP7 or HA-CSN5 in MDA-MB-231 cells. **C** PD-L1 expression in shCtrl and shPSMD14 MDA-MB-231 cells following CSN5 overexpression. **D-E** CHX chase analysis of PD-L1 half-life following USP7 (D) or CSN5 (E) overexpression in MDA-MB-231 cells with shCtrl or shPSMD14. **F-G** EdU and CCK-8 assays in BT-549 cells treated with Vector, Myc-PSMD14, or Myc-PSMD14 + si-PD-L1. Data are presented as mean ± SD (*n =* 3). **P* < 0.05, ***P* < 0.01, ****P* < 0.001, *****P* < 0.0001.

**Supplementary Figure 5**


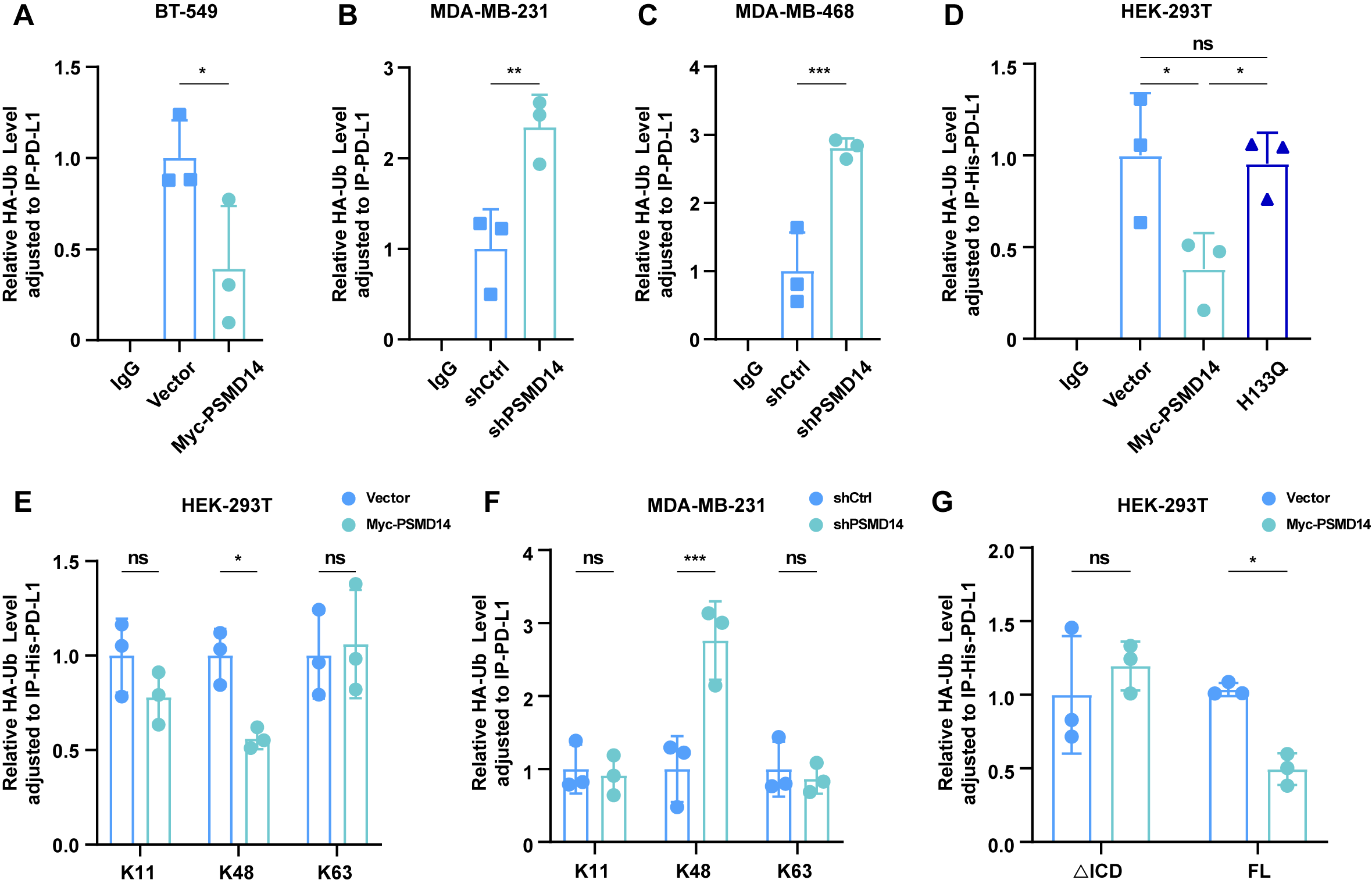


**Fig. S5 Quantitative analysis of ubiquitination assays.** **A-G** Quantification of PD-L1 ubiquitination corresponding to Fig. 4. Ubiquitination levels were normalized to immunoprecipitated PD-L1. ns, not significant, **P* < 0.05, ***P* < 0.01, ****P* < 0.001.

**Table S1. Analysis of the correlation between PSMD14 level and clinical characteristics in breast cancer patients.**

| **Characteristics** | **PSMD14 expression** | | ***χ²*** | ***P*-value** |
| --- | --- | --- | --- | --- |
|  | **Low (*n* = 31)** | **High (*n* = 59)** |  |  |
| **Age** |  |  | 0.113 | 0.737 |
| ≤ 50 | 12 | 25 |  |  |
| > 50 | 19 | 34 |  |  |
| **Lymph node** |  |  | 0.005 | 0.945 |
| Negative | 15 | 29 |  |  |
| Positive | 16 | 30 |  |  |
| **Tumor size** |  |  | 1.575 | 0.210 |
| < 2cm | 13 | 17 |  |  |
| ≥ 2cm | 18 | 42 |  |  |
| **Subtype** |  |  | 1.361 | 0.499 |
| ER positive | 6 | 7 |  |  |
| HER-2 positive | 6 | 9 |  |  |
| TNBC | 19 | 43 |  |  |
| **Histological grade** |  |  | 2.549 | 0.110 |
| I-II | 17 | 22 |  |  |
| III | 14 | 37 |  |  |
| **TNM stage** |  |  | 1.062 | 0.588 |
| I | 8 | 10 |  |  |
| II | 19 | 39 |  |  |
| III | 4 | 10 |  |  |
| **Ki-67** |  |  | 1.274 | 0.259 |
| ≤ 30% | 12 | 16 |  |  |
| > 30% | 19 | 43 |  |  |
| **Immunotherapy** |  |  | 1.097 | 0.295 |
| Yes | 14 | 20 |  |  |
| No | 17 | 39 |  |  |

|  |
| --- |

**Table S2. Analysis of the correlation between PSMD14 level and clinical characteristics in breast cancer patients treated with immunotherapy.**

| **Characteristics** | **PSMD14 expression** | | ***χ²*** | ***P*-value** |
| --- | --- | --- | --- | --- |
|  | **Low (*n* = 14)** | **High (*n* = 20)** |  |  |
| **Age** |  |  | 4.371 | 0.037 |
| ≤ 50 | 10 | 7 |  |  |
| > 50 | 4 | 13 |  |  |
| **Lymph node** |  |  | 0.215 | 0.643 |
| Negative | 6 | 7 |  |  |
| Positive | 8 | 13 |  |  |
| **Tumor size** |  |  | 0.578 | 0.447 |
| < 2cm | 2 | 5 |  |  |
| ≥ 2cm | 12 | 15 |  |  |
| **Histological grade** |  |  | 0.215 | 0.643 |
| I-II | 6 | 7 |  |  |
| III | 8 | 13 |  |  |
| **TNM stage** |  |  | 1.719 | 0.423 |
| I | 2 | 1 |  |  |
| II | 10 | 13 |  |  |
| III | 2 | 6 |  |  |
| **Ki-67** |  |  | 3.283 | 0.070 |
| ≤ 30% | 6 | 3 |  |  |
| > 30% | 8 | 17 |  |  |
| **PD-L1 CPS** |  |  | 1.561 | 0.211 |
| CPS<10 | 10 | 10 |  |  |
| CPS≥10 | 4 | 10 |  |  |
| **Responder** |  |  | 4.371 | 0.037 |
| Yes | 10 | 7 |  |  |
| No | 4 | 13 |  |  |

**Table S3. Targeting Sequence of shRNAs and siRNAs**

| **Gene Name** | **Species** | **Targeted Sequence** |
| --- | --- | --- |
| PSMD14-sh1 | Homo sapiens | CAAGCCATCTATCCAGGCATT |
| PSMD14-sh1 | Mus musculus | CTATGGAAGTTATGGGTCTAA |
| PSMD14-sh2 | Mus musculus | CGTCAGAGTGATTGATGTGTT |
| PSMD14-sh3 | Mus musculus | CGGACTAAACAGACATTATTA |
| siPD-L1#1 | Homo sapiens | CACAUCCUCCAAAUGAAAGTT |
| siPD-L1#2 | Homo sapiens | CAUUUGCUGAACGCAUUUATT |

**Table S4. Antibodies and reagents**

| **Antibody** | **Source** | **Identifier** |
| --- | --- | --- |
| PSMD14 | Proteintech | Cat# 12059-1-AP |
| PSMD14 | Abcam | Cat# ab109130 |
| PSMD14 | Cell Signaling Technology | Cat# D18C7 |
| PD-L1 | Proteintech | Cat# 66248-1-Ig |
| PD-L1 | Abcam | Cat# ab205921 |
| PD-L1 | Abways | Cat# CY5980 |
| USP7 | GenuIN BIOTECH | Cat# 61216 |
| CSN5 | GenuIN BIOTECH | Cat# 1599 |
| β-Tubulin | Sercicebio | Cat# GB15140 |
| His-tag | Proteintech | Cat# 66005-1-Ig |
| HA-tag | MCE | Cat# YA3393 |
| Myc-tag | MCE | Cat# HY-P80232 |
| HRP, Goat Anti-Mouse IgG | Abbkine | Cat# A21010 |
| HRP, Goat Anti-Rabbit IgG | Abbkine | Cat# A21020 |
| HRP, Mouse Anti-Rabbit IgG LCS | Abbkine | Cat# A25022 |
| Normal Rabbit IgG | Cell Signaling Technology | Cat# 2729 |
| FITC anti-human CD8 | Elabscience | Cat# E-AB-F1110C |
| PE anti-human/mouse Granzyme B | Biolegend | Cat# 372208 |
| PE anti-human CD274 | 4A Biotech | Cat# FHP274 |
| APC anti-human IFN-γ | Elabscience | Cat# E-AB-F1196E |
| Cell stimulation and Protein Transport Inhibitor Kit | Elabscience | Cat# E-CK-A091 |
| Foxp3/Transcription Factor Staining Kit | Elabscience | Cat# E-CK-A108 |
| Purified Anti-Mouse CD16/32 Antibody | Elabscience | Cat# E-AB-F0997A |
| Zombie NIR™ Fixable Viability Kit | Biolegend | Cat# 423106 |
| Violet 450 anti-mouse CD45 | Elabscience | Cat# E-AB-F1136Q |
| PerCP/Cyanine5.5 anti-mouse CD3 | Biolegend | Cat# 100217 |
| FITC anti-mouse CD8a | Elabscience | Cat# E-AB-F1104C |
| APC anti-mouse IFN-γ | Elabscience | Cat# E-AB-F1101E |
| FITC anti-mouse CD4 | Elabscience | Cat# E-AB-F1097C |
| APC anti-mouse CD25 | Biolegend | Cat# 101909 |
| PE anti-mouse FOXP3 | Biolegend | Cat# 320007 |
| FITC anti-mouse CD11b | Elabscience | Cat# E-AB-F1081C |
| PE anti-mouse Ly-6G/Ly-6C (Gr-1) | Biolegend | Cat# 108407 |
| 647 anti-mouse CD274 | Elabscience | Cat# E-AB-F1132M |
| PE/Cy7 Anti-Mouse MHC I (H-2Kd) | Elabscience | Cat# AN00429H |
| PE anti-mouse F4/80 | Biolegend | Cat# 123109 |
| PE/Cy7 anti-mouse CD206 | Biolegend | Cat# 141719 |
| APC anti-mouse CD86 | Biolegend | Cat# 105011 |
| PerCP/Cyanine5.5 anti-mouse MHC II | Elabscience | Cat# E-AB-F0990J |

**Table S5 Primer sequences used for qRT-PCR**

| **Gene Name** | **Species** | **Primer Sequence (5’ to 3’)** |
| --- | --- | --- |
| CD274-F | Homo sapiens | TGCCGACTACAAGCGAATTACTG |
| CD274-R | Homo sapiens | CTGCTTGTCCAGATGACTTCGG |
| GAPDH-F | Homo sapiens | AGGGCTGCTTTTAACTCTG |
| GAPDH-R | Homo sapiens | CTGGAAGATGGTGATGGG |
